# Pharyngeal pathology in a mouse model of oculopharyngeal muscular dystrophy is associated with impaired basal autophagy in myoblasts

**DOI:** 10.3389/fcell.2022.986930

**Published:** 2022-10-14

**Authors:** Yu Zhang, Christopher Zeuthen, Carol Zhu, Fang Wu, Allison T. Mezzell, Thomas J. Whitlow, Hyojung J. Choo, Katherine E. Vest

**Affiliations:** ^1^ Department of Molecular Genetics, Biochemistry and Microbiology, University of Cincinnati College of Medicine, Cincinnati, OH, United States; ^2^ Department of Cell Biology, Emory University School of Medicine, Atlanta, GA, United States

**Keywords:** oculopharyngeal muscular dystrophy, muscular dystrophy, dysphagia, PABPN1, satellite cells, craniofacial muscles, autophagy

## Abstract

Oculopharyngeal muscular dystrophy (OPMD) is a late-onset dominant disease that primarily affects craniofacial muscles. Despite the fact that the genetic cause of OPMD is known to be expansion mutations in the gene encoding the nuclear polyadenosine RNA binding protein PABPN1, the molecular mechanisms of pathology are unknown and no pharmacologic treatments are available. Due to the limited availability of patient tissues, several animal models have been employed to study the pathology of OPMD. However, none of these models have demonstrated functional deficits in the muscles of the pharynx, which are predominantly affected by OPMD. Here, we used a knock-in mouse model of OPMD, *Pabpn1* ^*+/A17*^, that closely genocopies patients. In *Pabpn1* ^*+/A17*^ mice, we detected impaired pharyngeal muscle function, and impaired pharyngeal satellite cell proliferation and fusion. Molecular studies revealed that basal autophagy, which is required for normal satellite cell function, is higher in pharynx-derived myoblasts than in myoblasts derived from limb muscles. Interestingly, basal autophagy is impaired in cells derived from *Pabpn1* ^*+/A17*^ mice. *Pabpn1* knockdown in pharyngeal myoblasts failed to recapitulate the autophagy defect detected in *Pabpn1* ^*+/A17*^ myoblasts suggesting that loss of PABPN1 function does not contribute to the basal autophagy defect. Taken together, these studies provide the first evidence for pharyngeal muscle and satellite cell pathology in a mouse model of OPMD and suggest that aberrant gain of PABPN1 function contributes to the craniofacial pathology in OPMD.

## Introduction

Oculopharyngeal muscular dystrophy (OPMD) is an autosomal dominant disease characterized by progressive dysphagia, ptosis, and upper limb weakness (Victor et al., 1962; Fried et al., 1975). While rare in the general population (1:100,000), large clusters exist in subgroups like French Canadians (1:1000) and Bukhara Jews (1:600) (Bouchard, 1997; Blumen et al., 2000). Dysphagia and loss of mobility decrease quality of life in individuals with OPMD and often patients succumb to dysphagia-associated aspiration pneumonia as the disease progresses. Despite the severe consequences for affected individuals, there are no pharmacologic treatments available and surgical interventions only temporarily alleviate some symptoms. The lack of drug development is in part because the molecular pathology of OPMD is still poorly understood.

OPMD is caused by small trinucleotide repeat expansions in the gene encoding the polyadenosine [poly (A)] binding protein nuclear 1 (PABPN1) (Brais et al., 1998). Wild type PABPN1 encodes ten alanine residues at the amino terminus but in individuals with OPMD, the alanine tract is expanded to 11–18 alanine residues (Banerjee et al., 2013; Jouan et al., 2014; Richard et al., 2015). PABPN1 binds to the poly(A) tails in RNAs and is best known for facilitating RNA polyadenylation by interacting with poly(A) polymerase and other components of the polyadenylation machinery (Kuhn et al., 2009). In addition to its canonical function, PABPN1 mediates multiple functions in RNA metabolism including but not limited to regulating alternative polyadenylation, nuclear RNA export, and translation (Banerjee et al., 2013). Muscle from individuals with OPMD is characterized by the presence of intranuclear aggregates containing PABPN1, RNA, other RNA binding proteins, and heat shock proteins (Tome and Fardeau, 1980; Calado et al., 2000; Fan et al., 2003; Tavanez et al., 2009; Klein et al., 2016). Several studies have demonstrated that overexpression of expanded PABPN1 correlates with increased aggregation, cell death, and severe muscle pathology in animal models of OPMD (Hino et al., 2004; Davies et al., 2005; Mankodi et al., 2012). However, PABPN1 aggregates are found in a small percentage of myonuclei in both affected and unaffected muscles from individuals with OPMD (Tome and Fardeau, 1980; Uyama et al., 2000; Dion et al., 2005; Gidaro et al., 2013). Wild type PABPN1 aggregates have also been identified in the murine hypothalamus (Berciano et al., 2004). Thus, PABPN1 aggregates may play an indirect role in OPMD pathology. Steady-state levels of PABPN1 are low in muscle, particularly in craniofacial muscles, relative to other tissues, suggesting that sequestration of PABPN1 in aggregates or impaired function of expanded PABPN1 may contribute to pathology by driving down already low levels of functional PABPN1 (Apponi et al., 2013; Phillips et al., 2018). This threshold model is supported by the fact that some studies suggest that loss of PABPN1 function contributes to OPMD pathology (Riaz et al., 2016; Vest et al., 2017).

The rare nature of OPMD makes human studies in age and sex matched individuals difficult, so animal models are essential to studying OPMD. However, generating an ideal OPMD model has been challenging. The most commonly studied mouse model, known as A17.1, contains a transgene encoding Ala17-PABPN1 under the control of a skeletal muscle actin promoter (A17.1 mice) (Davies et al., 2005). The A17.1 mice overexpress Ala17-PABPN1 at levels that are 10 to 30-fold above wild type levels (Randolph et al., 2014), contain high numbers of intranuclear PABPN1 aggregates, and develop severe wasting and functional deficits in limb muscles (Davies et al., 2005; Davies et al., 2006; Davies et al., 2010). The A17.1 mouse is an excellent model for identifying candidate drugs to remove aggregates and many of these do indeed resolve the muscle phenotypes in this model (Davies et al., 2005; Davies et al., 2006; Davies et al., 2010; Malerba et al., 2019b). However, PABPN1 aggregates are detected in a much higher percentage of myonuclei in A17.1 mice than in biopsies from individuals with OPMD, likely due to overexpression of the constitutively active A17.1 transgene. PABPN1 overexpression is a confounding factor for examining molecular pathology as elevated levels of even wild type PABPN1 can impact function (Banerjee et al., 2013) and corresponding Ala10-PABPN1 overexpressing controls are rarely used in A17.1 studies.

We previously developed an alternative mouse model of OPMD that represents the closest available genocopy to humans (Vest et al., 2017). These mice, termed *Pabpn1* ^*+/A17*^, contain a single expanded *Pabpn1* allele under the control of the native promoter while the second *Pabpn1* allele is wild type. The *Pabpn1* ^*+/A17*^ mice express native levels of Ala17-PABPN1 in all tissues including lower levels in muscle relative to other tissues. The mild muscular phenotypes observed in *Pabpn1* ^*+/A17*^ mice render them an ideal model for studying the molecular pathology of OPMD as there are no confounding factors resulting from PABPN1 overexpression or from severely damaged muscle. Using this model, we identified a much milder effect of Ala17-PABPN1 on alternative polyadenylation than previously identified, with only small subsets of genes being affected. We also detected overlapping phenotypes in *Pabpn1* ^*+/A17*^ mice and heterozygous PABPN1 knockout (*Pabpn1* ^*+/∆*^) mice, suggesting that loss of PABPN1 function contributes to OPMD. However, this study did not include analysis of pharyngeal muscles.

Pharyngeal muscle pathology is the most significant and prognostic symptom for the majority of individuals with OPMD (Youssof, 2016; Kroon et al., 2021). However, studies probing pharyngeal pathology in animal models are limited. A previous study of the A17.1 mouse revealed mild histopathology but no functional pathology in pharyngeal muscles. Rather, overexpression of Ala10-PABPN1 was found to be protective against age-related dysphagia while both wild type and A17.1 mice showed similar age-related dysphagia (Randolph et al., 2014). Pharyngeal muscle is distinct from muscles of the limb and trunk in both embryonic origin and adult muscle characteristics (Sambasivan et al., 2009; Sambasivan et al., 2011; Randolph et al., 2015; Kim et al., 2022). Skeletal muscle stem cells, termed satellite cells, are typically quiescent in limb muscle and must be activated by hepatocyte growth factor (HGF) and other hormones originating from immune and mesenchymal cells in the injured muscle niche (Tatsumi et al., 1998; Miller et al., 2000; Tajbakhsh, 2009; Kim et al., 2022). However, in pharyngeal muscles, the satellite cell pool proliferates in the absence overt muscle damage (Randolph et al., 2015; Kim et al., 2022). Although some studies in OPMD patient tissues have suggested pharyngeal satellite cell involvement (Perie et al., 2006), no studies in animal models have probed the effect of alanine expanded PABPN1 on satellite cell function *in vivo*. Thus, molecular pathology affecting satellite cells in OPMD remains poorly characterized.

Here, we studied the pharyngeal muscle pathology of *Pabpn1* ^*+/A17*^ mice. We found that at 6 months of age, *Pabpn1* ^*+/A17*^ mice exhibit detectable dysphagia along with mild cricopharyngeal histopathology. Interestingly, we detected a significant decline in pharyngeal satellite cell proliferation and fusion in *Pabpn1* ^*+/A17*^ mice, which was not detected in limb muscles. We also detected a significant defect in basal autophagy in cultured pharyngeal satellite cell-derived myoblasts from *Pabpn1* ^*+/A17*^ mice while no defect was detected in limb-derived *Pabpn1* ^*+/A17*^ myoblasts. This autophagy defect is correlated with a decline in levels of *Beclin1* mRNA, which encodes a critical component of autophagosome formation (Cao and Klionsky, 2007). Autophagy defects and decreased *Beclin1* levels were not detected after knocking down *Pabpn1*, suggesting that aberrant gain of function may contribute to autophagy phenotypes in *Pabpn1* ^*+/A17*^ mice. Taken together, these data indicate that pharynx-specific phenotypes including decreased satellite cell proliferation, impaired basal autophagy, and decreased levels of *Beclin1* may contribute to OPMD pathology. Importantly, these phenotypes would not be detected in any other mouse models of OPMD as none express alanine expanded PABPN1 in satellite cells, which further cements the utility of the *Pabpn1* ^*+/A17*^ mouse model in studying OPMD.

## Materials and methods

### Mice

Multiple mouse models were used to analyze the *in vivo* effects of alanine-expanded PABPN1. *Pabpn1* ^*+/A17*^ knock-in mice were generated as described previously (Vest et al., 2017) and were genotyped by PCR (Supplementary Table S1). C57BL/6J mice (Jax000664), B6.Cg-*Pax7* ^*tm1(cre/ERT2)Gaka*^/J mice (Jax017763), B6.Cg-*Gt(ROSA)26Sor* ^*tm9(CAG-tdTomato)Hze*^/J mice (Jax007909) were purchased from Jackson Laboratories. Six-month-old mice or 12-month-old mice were used as noted in figure legends. To obtain Pabpn1^+/A17^ knock-in; Pax7 ^CreERT2/+^; Rosa ^tdTomato/+^ (Pabpn1^+/A17^-Pax7 Cre^ERT2^-tdTomato) mice, heterozygous Pax7 ^CreERT2/+^;Rosa ^tdTomato/+^ male mice were crossed with Pabpn1^+/A17^ knock-in female mice. To activate Cre recombinase to label satellite cells with red fluorescence (tdTomato), tamoxifen at 1 mg (Sigma-Aldrich) per 10 g body weight was injected intraperitoneally once daily for 5 days. Experiments were performed in accordance with approved guidelines and ethical approval from Emory University’s Institutional Animal Care and Use Committee (PROTO201700233), University of Cincinnati’s Institutional Animal Care and Use Committee (Protocol # 21-10-14-01), and in compliance with the National Institutes of Health.

### Mouse behavioral assays

Dysphagia in mice was measured by food/water consumption and lick assay (Randolph et al., 2014; Kim et al., 2022). To measure individual food and water consumption, each mouse was housed in a single cage. The water bottle (a sipper tube with 50 ml conical tube) and dry food (Laboratory Rodent Diet 5001) were weighed and placed in each cage every day for 4 days. Body weight for each mouse was measured at the same time for 4 days. On the 4th day, water bottles were removed from each cage for 16 h. After 16 h, water bottles were replaced and lick episodes were recorded starting about 30 s after reintroducing water. Video play speed was slowed to 0.1X using the iMovie app (Apple) and the tongue protrusions/second for each mouse were counted. For forelimb grip strength, a grip strength meter (Columbus Instruments) was first set to zero and then mice were allowed to grab the front wire of the meter with forelimbs. The mouse was slowly pulled back from the meter by the tail and force was measured at the time the mouse released its grip. Each mouse was tested a total of three times and the average of all three readings was used for the final measurement. Mice were assayed for endurance and coordination by rotarod test as previously described (Yang et al., 2014). Briefly, mice were trained on the rotarod (Rotamex, Columbus Instruments) at 5 rpm for 10 min each over 3 days. For testing, mice were placed on the rotarod and the speed was accelerated from 0 to 40 rpm over 3 min. Latency to fall (LTF) was recorded automatically by the instrument and testing was stopped after 6 min. Each mouse was tested a total of three times and the average of all LTF measurements was compared.

### Histology and immunofluorescence

To identify the effects of alanine-expanded PABPN1 on muscles *in vivo*, histologic analysis was performed. Pharyngeal tissue dissection was performed as described previously (Randolph et al., 2014; Kim et al., 2022). Briefly, we dissected pharyngeal tissue extending from the soft palate caudally to the trachea and esophagus. Tibialis anterior (TA) or quadriceps muscles were dissected as previously stated (Vest et al., 2017). Muscle tissues were frozen in Tissue-Tek O.C.T (Sakura Finetek United States) or Tissue Freezing Medium (Triangle Biomedical Sciences) by floating the mold on top of super-cooled 2-methylbutane over liquid nitrogen and then stored at −80°C. Tissue cross sections of 10 μm thickness were collected every 100 μm for pharyngeal muscles and every 200 μm for TA or quadriceps muscles using a Leica CM1850 cryostat. Muscle sections were stained with hematoxylin and eosin (H&E) to observe basic morphology and to measure the cross-sectional area and count central nucleated fibers. To mark muscle membrane, we immunostained with an antibody to laminin. Sections were incubated with blocking buffer (5% goat serum, 5% donkey serum, 0.5% BSA, 0.25% Triton-X 100 in PBS) for 1 h at room temperature and then incubated overnight at 4°C with primary anti-Laminin (Sigma Aldrich) or isotype control diluted 1:300 in blocking buffer overnight at 4 °C in blocking buffer. The following day, sections were washed three times with washing buffer (0.2% Tween-20 in PBS) and incubated with FITC-conjugated anti-rabbit antibody (1:500) for 1 h at room temperature. Nuclei were then stained with 4’,6-diamidino-2-phenylindole (DAPI) (1 μg/ml) and mounted using Vectashield (Vector Labs). Sections were imaged on a Revolve Echo widefield fluorescence microscope at ×10 or ×20 magnification. For *in vivo* SC fusion assay, cross-sectioned muscles from *Pabpn1* ^*+/A17*^ *-Pax7 Cre* ^*ERT2*^ *-tdTomato* mice and controls were stained with laminin to distinguish muscle fibers. After image acquisition with consistent imaging conditions, images from the red channel (tdtomato) were converted to grayscale and we measured the mean gray value (range from 0 (black) to 255 (white)) of cross-sectioned muscle fibers using ImageJ. For injured tibialis anterior muscles, we imaged H&E-stained muscle sections at ×10 magnification and cross-secitonal area (in µm^2^) was measured using ImageJ.

### Bromodeoxyuridine labeling and flow cytometry

To compare the SC proliferation in pharyngeal and quadriceps muscles *in vivo*, Bromo-2′-deoxyuridine (BrdU) assays were performed as previously described (Randolph et al., 2015; Kim et al., 2022). Three-month-old *Pabpn1* ^*+/A17*^ and *Pabpn1* ^*+/+*^ male mice were given intraperitoneal injections of 10 µg BrdU (Sigma-Aldrich) per gram body weight every 12 h for a total of 48 h. An additional 0.8 mg/ml BrdU was provided in drinking water along with 5% sucrose to encourage consumption. Pharyngeal or quadriceps muscles dissected and minced and then resuspended in Dulbecco’s modified Eagle’s medium (DMEM). Minced muscles were digested in 800U/ml collagenase II (Gibco) for 1 h. After washing, muscles were digested again using 100U/ml collagenase II (Gibco) and 1.1U/ml dispase (Gibco) for 30 min as described (Liu et al., 2015). Mononucleated cells were then isolated from remaining connective tissue and other debris using a 100 µm filter. To assess proliferation, mononucleated cells were stained with antibodies to CD31, CD45, Sca1, and α-7 integrin (Supplementary Table S2). Subsequently cells were labeled for BrdU using a FITC-BrdU flow kit in accordance with the manufacturer’s instructions (BD Biosciences). Proliferating SCs were collected according to the following sorting criteria: CD31^−^/CD45^−^/Sca1^−^/Intergrin7α^+^/BrdU^+^.

### *In vivo* muscle injury

Induced muscle injury was used to study satellite cell behavior in limb muscles (Choo et al., 2017). Injury was induced in tibialis anterior (TA) muscles as previously described. Briefly, mice were anesthetized with ketamine hydrochloride (80 mg/kg) and xylazine (5 mg/kg) administered by intraperitoneal injection and 27G needle. TA muscles were then injected longitudinally with 25 µl of 1.2% BaCl_2_ using a Hamilton syringe. Mice received two subcutaneous injections of 0.1 mg/kg buprenorphine every 12 h for analgesia. TA muscles were collected for analysis 7 days after inducing injury.

### Myoblast isolation and cell culture

*In vitro* experiments were performed using primary myoblasts isolated *Pabpn1* ^*+/A17*^ and *Pabpn1* ^*+/+*^ mice. Myoblasts were isolated as previously described (Choo et al., 2017; Kim et al., 2022). Mononucleated cells were dissected from pharyngeal muscles or bulk hindlimb muscles, minced, and digested with 0.1% Pronase (EMD Millipore) in DMEM with 25 mM HEPES buffer for 1 h at 37°C with gentle stirring. Digested muscle tissue was rinsed with DMEM containing 10% FBS (Cytiva Life Sciences), triturated with a 25 ml serological pipette, and filtered using a 100 µm Steriflip filter (EMD Millipore). Pharyngeal myoblasts were plated on collagen-coated dishes in Ham’s F10 (Thermo Fisher Scientific), 20% FBS, 100 μg/ml penicillin/streptomycin with 5 ng/ml basic fibroblast growth factor (PeproTech). If necessary, we used pre-plating to remove non-myoblast cell types. After trypsinization, cells were incubated in non-collagen coated tissue culture dishes to capture rapidly adhering cells for 1 h before being replated on collagen-coated plates. Limb myoblasts were isolated using magnetic activated cell sorting (MACS). Residual blood cells were lysed with ammonium-chloride-potassium (ACK) buffer (Gibco). Cells were washed in 2% bovine serum albumin (BSA) and labeled with biotin-conjugated antibodies to CD31, CD45, and Sca1 in the Miltenyi Satellite Cell isolation kit (Miltenyi Biotec). CD31^+^/CD45^+^/Sca1^+^ cells were isolated with magnetic streptavidin-coated beads and unbound CD31^−^/CD45^−^/Sca1^−^ flow-through cells were collected as the myoblast fraction. Limb myoblasts were grown under the same conditions as were pharyngeal myoblasts.

### *In vitro* assay of proliferation and viability

Proliferation was assayed in myoblasts using the click-IT EdU labeling kit (Invitrogen) according to the manufacturer’s instructions. Briefly, cells were seeded at equal density and allowed to grow for 24 h. Cells were then incubated in 10 µM EdU for 3 hours at 37°C before staining, nuclear labeling with DAPI, and visualizing by fluorescence microscopy. Proliferating cells were quantified as percentage of EdU positive normalized to DAPI stained nuclei. Cell counting was performed using DAPI labeling after seeding equal cell density and allowing cells to grow for 48 h. Cell viability was determined using the RealTime-Glo MT Cell Viability Assay (Promega) according to the manufacturer’s instructions for endpoint assay. Briefly, cells were seeded at equal density and allowed to grow for 24 h. Cells were then treated with the RealTime-Glo reagent for 1 h before reading luminescence on a BioTek plate reader.

### *In vitro* autophagy

Starvation conditions were used to induce autophagy in primary myoblasts isolated from pharyngeal or limb muscles. Cells were grown to approximately 60% confluence and growth medium was washed away and replaced with Hank’s balanced salt solution (HBSS) modified with sodium bicarbonate. Cells were incubated in HBSS or normal growth medium (Ham’s F10 + 20% FBS with 100 μg/ml penicillin/streptomycin and 5 ng/ml basic fibroblast growth factor) with and without added chloroquine (100 µM final concentration) for 4 h at 37°C. Cells were harvested in radioimmunoprecipitation (RIPA) buffer (Thermo Fisher) and processed for immunoblotting.

### Immunoblotting

Autophagy was quantified by measuring the ratio of LC3-II/I as detected by immunoblot. Growth medium was aspirated and cells were washed twice with PBS before being scraped into RIPA buffer (Thermo Fisher) with added Protease Inhibitor tablets (Pierce). Lysates were sonicated at 40% output for 10 s three times each, incubated on ice for 30 min, and centrifuged at 21,000 *x g* to remove insoluble debris. Lysates were boiled in Laemmeli buffer (Bio-Rad) and separated on BioRad Mini-Protean TGX Stain-free 4–20% polyacrylamide gradient gels. After gels were transferred to nitrocellulose, membranes were blocked with 5% milk suspended in Tris buffered saline containing 0.1% Tween-20 (TBS-T) for 1 h at room temperature and incubated in primary antibody suspended in TBS-T (Supplementary Table S1) overnight at 4°C. Blots were then washed in TBS-T and incubated in horseradish peroxidase (HRP) conjugated secondary antibody diluted 1:10,000 in 5% milk in TBS-T for 1 h at room temperature. After washing, target proteins were detected using SuperSignal West Pico PLUS Chemiluminescent Substrate (Thermo Fisher Scientific). Blots were imaged on a ChemiDoc Touch Imaging System and bands were quantified on Image Lab software (BioRad).

### RNA isolation and quantitative reverse transcriptase-PCR

Steady state-RNA levels and RNA immunoprecipitations were quantified by qRT-PCR. Total RNA was isolated using TRIzol (Invitrogen) according to the manufacturer’s instructions. For each sample, 500 ng of RNA was converted to complimentary DNA (cDNA) using the Maxima First Strand cDNA Synthesis Kit with dsDNase (Thermo Fisher). Amplification was performed using SYBR Select Master Mix (Applied Biosystems) on a QuantStudio 3 Real Time PCR system (Applied Biosystems). All primer sequences are listed in Supplementary Table S2. PCR results were quantified using the comparative Ct method (Livak and Schmittgen, 2001) using *Gapdh* as a normalizer.

### RNA-immunoprecipitation

PABPN1 binding to RNA targets was determined by RNA immunoprecipitation (RIP). Myoblasts were lysed in ice old passive lysis buffer (Promega) supplemented with protease inhibitor (Pierce) and Rnasin (Promega). Lysates were pipetted 20 times to break cells and centrifuged at 15,000 x g for 15 min at 4°C. Protein concentration in resulting supernatants were determined by Bradford assay and 10% of lysate was removed for input fractions. Remaining lysate was subjected to pre-clearing. Briefly, Protein A Dynabeads (Invitrogen) were washed twice with PBS + 0.1% Tween-20 and twice with NT2 buffer (10 mM Tris-HCl, pH 7.4, 150 mM NaCl, 1 mM MgCl_2_, 0.05% Nonidet P-40, 50 µM dithiothreitol, and 100 units/ml Rnasin), lysates were then applied and rotated end-over-end at room temperature for 30 min. Lysates were then incubated with 1 µg anti-PABPN1 antibody or 1 µg non-specific rabbit IgG (Supplementary Table S1), rotating end-over-end, at 4°C overnight. Resulting antibody-complexed lysates were then incubated with pre-washed Protein A beads and rotated end-over-end for 1 h at room temperature. After removing supernatant, beads were washed five times with NT2 buffer for 5 min each. Bound RNA was then isolated directly from beads using TRIzol (Invitrogen). RNA was converted to cDNA and analyzed by qRT-PCR using primers targeting *Becn1* RNA and the positive control RNA *Neat1*.

### Polyadenosine [poly (A)] binding protein nuclear 1 knockdown and polyadenosine [poly (A)] binding protein nuclear 1 overexpression

To determine if loss of PABPN1 function contributes to autophagy phenotypes, we used duplexed siRNA (DsiRNA, Integrated DNA Technologies) to knock down *Pabpn1* in primary myoblasts isolated from pharyngeal muscles. Based on previous experiments to identify DsiRNA with optimal knockdown capability (data not shown), cells were transfected with 100 nM *Pabpn1* DsiRNA (mm.Ri.Pabpn1.13.1) using Lipofectamine 3000 (Thermo Fisher) according to the manufacturer’s instructions. As a negative control, cells were transfected with either 100 nM negative control duplexed RNA (scr) or mock transfection mix (NT) containing water and lipofectamine 3000. To determine if wild type PABPN1 overexpression or wild type PABPN1 expression in combination with *Pabpn1* knockdown rescues the autophagy phenotype, we transfected cells with a pcDNA 3.1 plasmid encoding N-terminal FLAG-tagged human PABPN1 (FL-hsPABPN1) (Banerjee et al., 2019) with or without *Pabpn1* siRNA using lipofectamine 3000. As a control, cells were mock transfected with lipofectamine 3000 with no nucleic acids. In all cases, cells were harvested in RIPA or subjected to starvation conditions to induce autophagy 48 h after transfection. Immunoblotting and qRT-PCR were used to confirm knockdown and immunoblotting for the LC3 II/I ratio was used to measure autophagy.

### Statistical analyses

Statistical analysis was performed using Prism 9.0 for Mac OS (GraphPad). Statistical methods employed, ranges of *p* values, and sample size are indicated in figure legends. For all experiments except distribution of myofiber cross-sectional area (CSA), results are expressed as the mean ± standard error of the mean (SEM). Experiments were repeated at least three times unless otherwise noted in figure legends. Statistical testing was determined by normality test, such as the Shapiro-Wilk test and Kolmogorov-Smirnov test. If data showed normal distribution by the normality tests, statistical analysis was performed using Student’s t-test or 1-way or 2-way ANOVA as stated in the figure legends. If data showed non-normal distribution, we chose non-parametric statistical analysis as stated in the figure legends. *p* < 0.05 was considered statistically significant.

## Results

### *Pabpn1* ^*+/A17*^ mice exhibit pharynx-specific functional defects and histologic pharyngeal pathology

**FIGURE 1:**
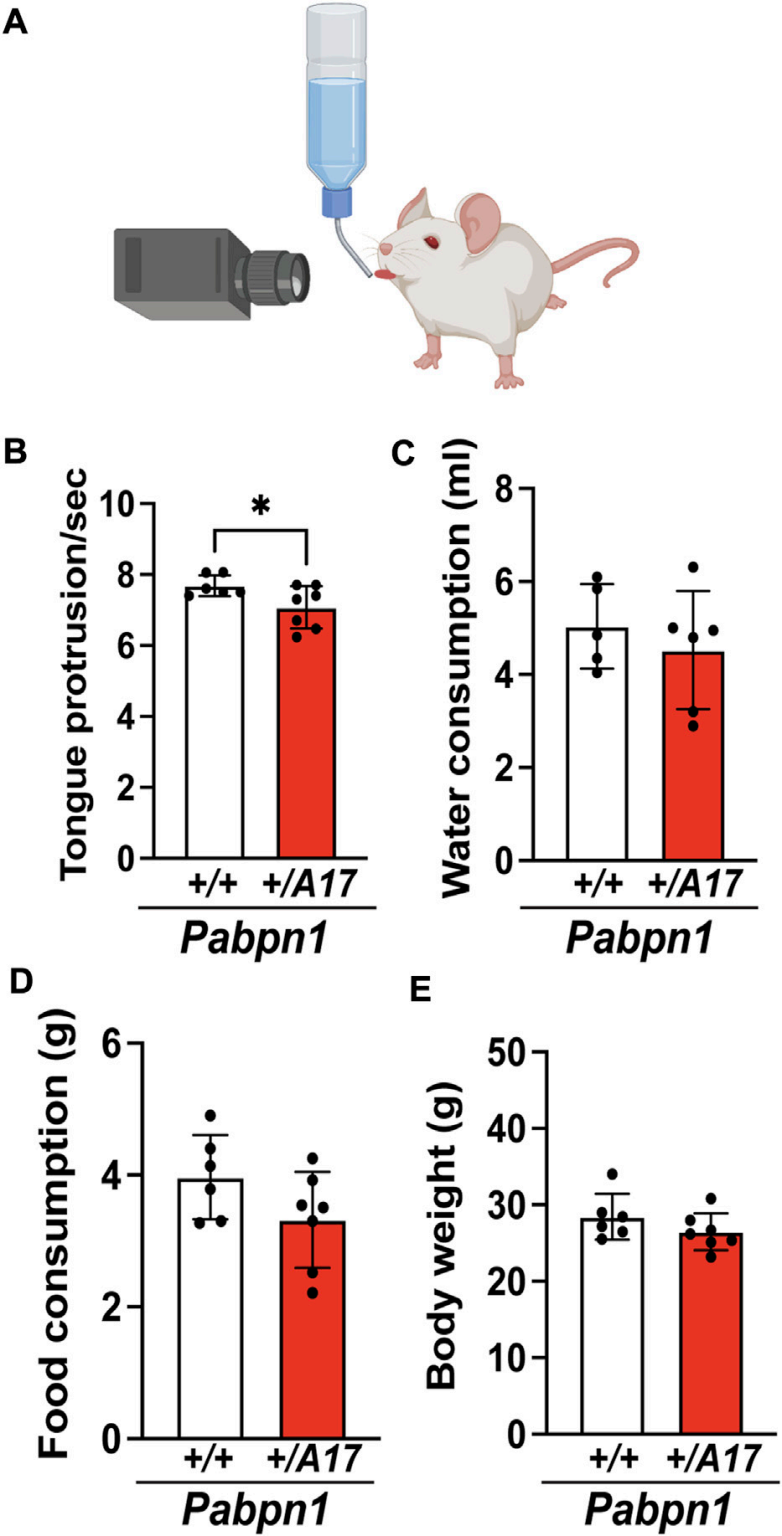
*Pabpn1* ^*+/A17*^ mice exhibit functional pharyngeal muscle defects. **(A)** Illustration of lick assay used to measure swallow function in mice. **(B)** Number of tongue protrusions per second is significantly decreased in *Pabpn1* ^*+/A17*^ mice as counted by video analysis of lick assay. **(C,D)** No difference of daily water consumption (ml) **(C)** and daily food consumption **(G) (D)** between *Pabpn1* ^*+/+*^ mice and *Pabpn1* ^*+/A17*^ mice were measured. **(E)** Body weight was not different between *Pabpn1* ^*+/+*^ mice and *Pabpn1* ^*+/A17*^ mice. Shown is mean ± SEM for *n* = 6–7 mice per genotype. Statistical significance was determined by Student’s t-test (*p < 0.05).

For the majority of individuals with OPMD, dysphagia is the earliest and most prognostic symptom (Youssof, 2016; Kroon et al., 2021). To determine if *Pabpn1* ^*+/A17*^ mice exhibit swallowing defects, we employed an indirect measure of swallowing speed and pharyngeal muscle function that calculates the rate at which the mouse tongue makes contact with a water bottle (Figure 1A). In our previous study, limb muscle pathology was detectable by 6 months of age in *Pabpn1* ^*+/A17*^ mice (Vest et al., 2017). Thus, we assayed lick rate in 6-month-old *Pabpn1* ^*+/A17*^ mice and detected an approximate 10% decrease in comparison to *Pabpn1* ^*+/+*^ control mice (Figure 1B). We detected no difference in water consumption between groups (Figure 1C) and a trend toward decreased food consumption in *Pabpn1* ^*+/A17*^ mice that was not significant (Figure 1D). Weight loss, another symptom of OPMD, was not detected in 6-month-old *Pabpn1* ^*+/A17*^ mice (Figure 1E). Considering that OPMD is an age-related disease, we assayed lick rate in 12-month-old mice and did not detect a defect in *Pabpn1* ^*+/A17*^ mice (Figure S1A). However, the lick rate in 12-month-old *Pabpn1* ^*+/+*^ control mice was slightly lower than in 6-month-old mice, which is consistent with previously reported age-related dysphagia in mouse models (Randolph et al., 2014). Taken together, these data suggest that at 6 months of age, *Pabpn1* ^*+/A17*^ mice exhibit weak dysphagia, which is consistent with the dysphagia experienced by humans at the onset of OPMD but the effect is lost with age.

**FIGURE 2:**
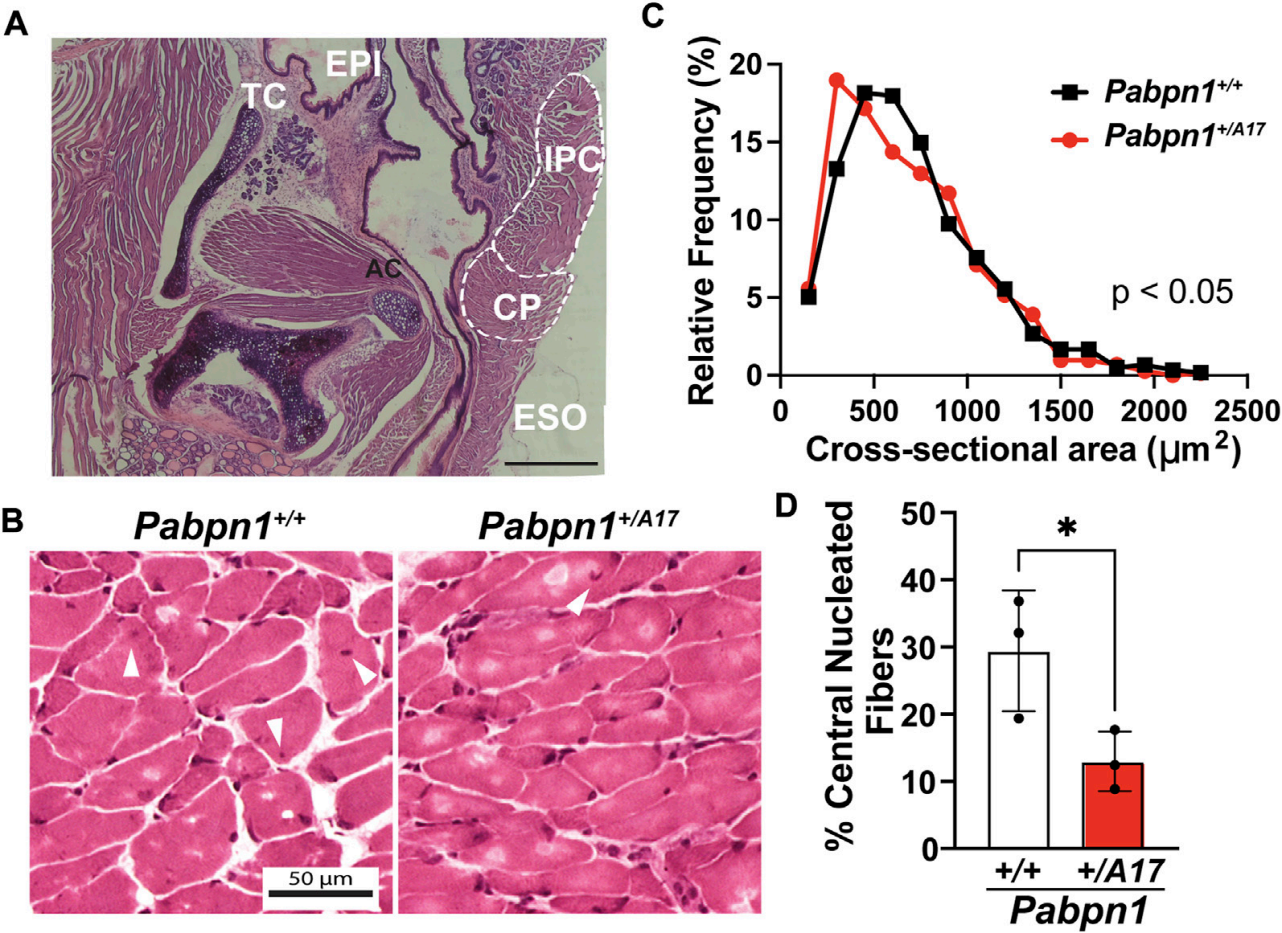
Pharyngeal muscles from *Pabpn1* ^*+/A17*^ mice contain smaller myofibers and evidence of impaired satellite cell function **(A)** Representative H&E-stained longitudinal section labeling anatomy of the laryngeal pharynx including cricopharyngeus (CP), esophagus (ESO), inferior pharyngeal constrictor (IPC), adenoid cartilage (AC), thyroid cartilage (TC), and epiglottis (EPI). Bar = 330 µm. **(B)** Representative H&E-stained CP muscle cross sections from *Pabpn1* ^*+/+*^ mice and *Pabpn1* ^*+/A17*^ mice. White arrowheads mark central nuclei. Bar = 50 µm. **(C)** Frequency distribution of CP cross sectional area (mm^2^) revealing a significant increase in small fibers in *Pabpn1* ^*+/A17*^ mice. Shown is frequency distribution of binned cross-sectional area data from *n* = 3 mice per genotype. Statistical significance was determined using non-parametric Kolmogorov-Smirnov test. **(D)** Percentage of central nucleated fibers was significantly decreased in *Pabpn1* ^*+/A17*^ mice. Shown is mean ± SEM for *n* = 3 mice. Statistical significance was determined using Student’s t-test (*p < 0.05).

To determine if dysphagia correlates with impaired pharyngeal muscle structure, we performed hematoxylin and eosin (H&E) staining on isolated pharyngeal muscles from 6-month-old *Pabpn1* ^*+/A17*^ mice and corresponding *Pabpn1* ^*+/+*^ control mice. The pharynx consists of a group of 7 separate muscles that function to coordinate swallowing (Randolph et al., 2014; Kim et al., 2022). In OPMD, the cricopharyngeal muscle, which resides just anterior to the esophageal and tracheal openings (Figure 2A), is frequently affected. Upon examination, the cricopharyngeus in *Pabpn1* ^*+/A17*^ mice did not exhibit any overt pathology (Figure 2B). However, quantification of myofiber cross-sectional area revealed an increase in myofibers with smaller cross-sectional area (Figure 2C). Taken together, the functional and histologic data suggest that at 6 months of age, *Pabpn1* ^*+/A17*^ mice exhibit mild pharyngeal muscle functional and histologic pathology, which may correspond to pre-symptomatic or early symptomatic individuals with OPMD.

Craniofacial muscles, including those of the pharynx, arise from unique embryonic origins (Tajbakhsh, 2009; Grimaldi and Tajbakhsh, 2021) and exhibit unique properties relative to the muscles of the trunk and limbs. In pharyngeal muscles, the typically quiescent satellite cell pool remains active under basal conditions (Randolph et al., 2015; Kim et al., 2022). One hallmark of recent satellite cell fusion is the existence of central nucleated fibers (Randolph et al., 2015). As expected, the cricopharyngeal muscles from 6-month-old *Pabpn1* ^*+/+*^ mice contained ∼25% central nucleated fibers (Figures 2B,D) while as previously reported the rectus femoris muscle from age-matched mice showed ∼3% central nucleated fibers (Vest et al., 2017). Interestingly, the cricopharyngeal muscles from *Pabpn1* ^*+/A17*^ mice contained half the number of central nucleated fibers (Figure 2D). Considering that central nucleated fibers arise from recently fused satellite cells, this result suggests that satellite cells in the cricopharyngeal muscles of *Pabpn1* ^*+/A17*^ mice may exhibit reduced activity.

Although OPMD primarily affects the craniofacial muscles, many affected individuals experience severe limb muscle weakness (Youssof, 2016). Our previous study indeed revealed a mild decrease in limb muscle cross-sectional area as well as the presence of several small myofibers. Here, we performed grip strength measurement and rotarod trials to determine if *Pabpn1* ^*+/A17*^ mice experience limb muscle weakness. We detected no significant difference in grip strength normalized to mouse weight in *Pabpn1* ^*+/A17*^ mice relative to controls (Supplementary Figure S1A), though the measurements for *Pabpn1* ^*+/A17*^ mice were much more variable than those for control mice. Similarly, we did not detect any significant change in latency to fall during rotarod trials (Supplementary Figure S1B). Taken together, these data suggest that, unlike previously studied mouse models of OPMD, *Pabpn1* ^*+/A17*^ mice exhibit functional pathology specific to pharyngeal muscles.

### Pharyngeal satellite cells are impaired in *Pabpn1* ^*+/A17*^ mice

**FIGURE 3:**
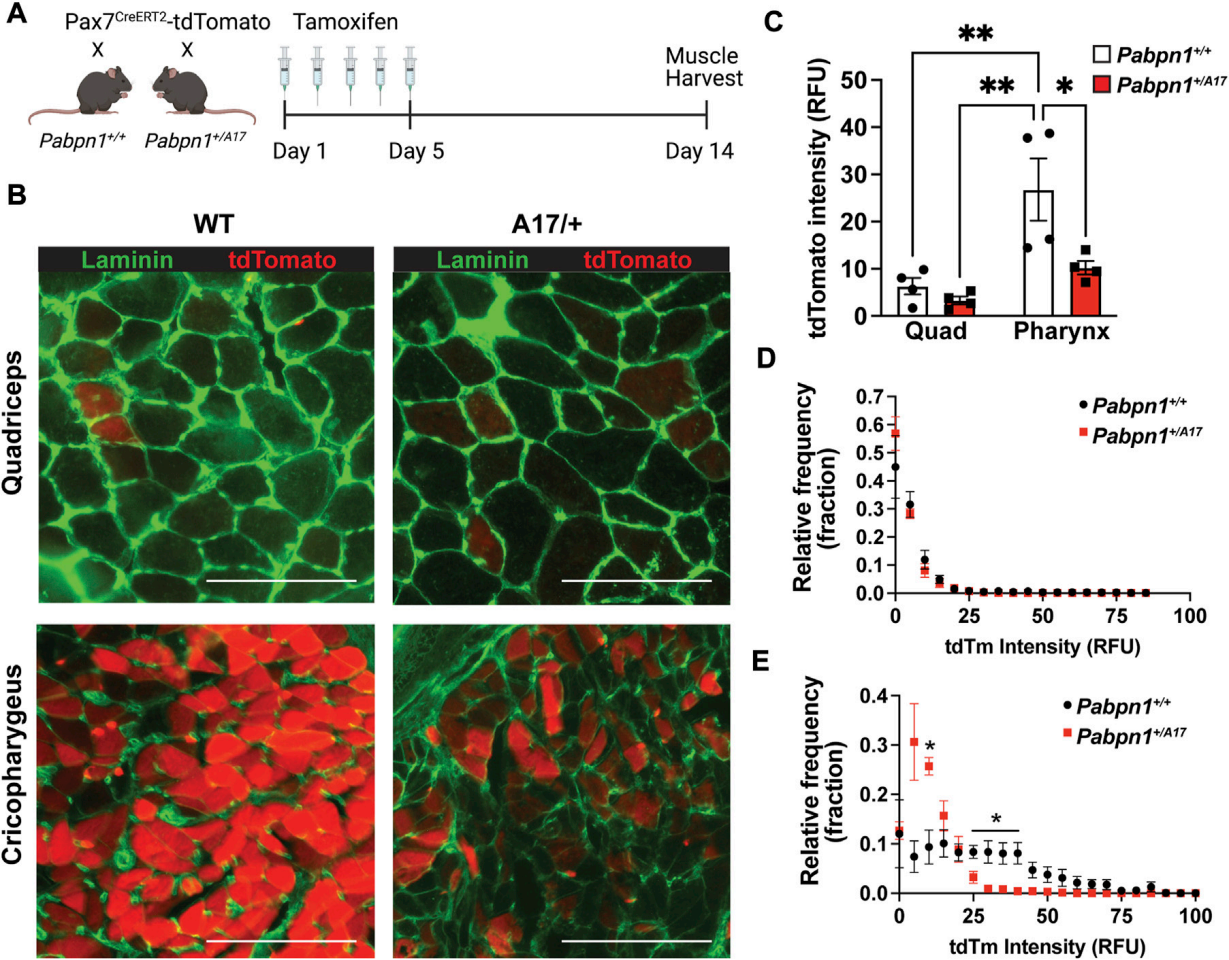
*Pabpn1* ^*+/A17*^ mice have impaired pharyngeal satellite cell fusion **(A)** Schematic of tamoxifen treatment plan for Pax7Cre^ERT2^-tdTomato mice crossed with control or *Pabpn1* ^*+/A17*^ mice. Mice were treated with tamoxifen for 5 days at 2.5 months of age and harvested at 6 months of age. **(B)** Representative images of cross-sectioned quadriceps or cricopharyngeus muscles showing fibers with recently fused satellite cells as tdTomato positive (red) and stained with laminin (green) to outline all fibers. Bar = 130 µm. **(C)** Average tdTomato intensity in relative fluorescence units (RFU) showing increased tdTomato intensity in cricopharyngeus (PH) relative to quadriceps (Quad) and a decrease in cricopharyngeal tdTomato intensity in *Pabpn1* ^*+/A17*^ relative to *Pabpn1* ^*+/+*^ mice. **(D)** Frequency distribution (fraction) of tdTomato intensity in myofibers in quadriceps muscles showing no change in *Pabpn1* ^*+/A17*^ mice. **(E)** Frequency distribution (fraction) of tdTomato intensity in myofibers in cricopharyngeus muscles showing increased number of fibers with low or no tdTomato signal in *Pabpn1* ^*+/A17*^ mice. Shown is mean ± SEM for *n* = 4 mice. Statistical significance was determined using two-way ANOVA (* *p* < 0.05, ** *p* < 0.01).

The decreased number of central nucleated fibers detected in *Pabpn1* ^*+/A17*^ mice suggests impaired satellite cell fusion. To assay satellite cell fusion, we crossed *Pabpn1* ^*+/A17*^ mice with *Pax7-Cre* ^*ERT2*^ *-tdTomato* mice, which encode an inducible satellite cell-specific tdTomato label (Choo et al., 2017). As adult limb muscles show low levels of satellite cell fusion (Pawlikowski et al., 2015), *Pabpn1* ^*+/A17*^ x *Pax7-Cre* ^*ERT2*^ *-tdTomato* mice were maintained for 3.5 months after tamoxifen injection (Figure 3A). When tdTomato labeled satellite cells fuse into a myofiber, the tdTomato protein can be detected in the cytoplasm. We compared cross-sections of cricopharyngeal muscle and quadriceps (rectus femoris) muscle from *Pabpn1* ^*+/A17*^ x *Pax7-Cre* ^*ERT2*^ *-tdTomato* mice and *Pabpn1* ^*+/+*^ x *Pax7-Cre* ^*ERT2*^ *-tdTomato* (Figure 3B)*.* While no change was detected in the average tdTomato intensity or the distribution of tdTomato intensity across myofibers in quadriceps muscle (Figures 3C,D), a significant decrease was detected in cricopharyngeal muscles of *Pabpn1* ^*+/A17*^ x *Pax7-Cre* ^*ERT2*^ *-tdTomato* mice (Figures 3C,E). The overall intensity of tdTomato was higher in cricopharyngeal muscles relative to quadriceps muscles, which is consistent with the high levels of basal satellite cell fusion detected in craniofacial muscles (Pawlikowski et al., 2015; Randolph et al., 2015; Kim et al., 2022). Taken together, these data indicate impaired satellite cell fusion in cricopharyngeal muscle from *Pabpn1* ^*+/A17*^ mice, which is consistent with the decrease in central nucleated fibers (Figure 2D).

**FIGURE 4:**
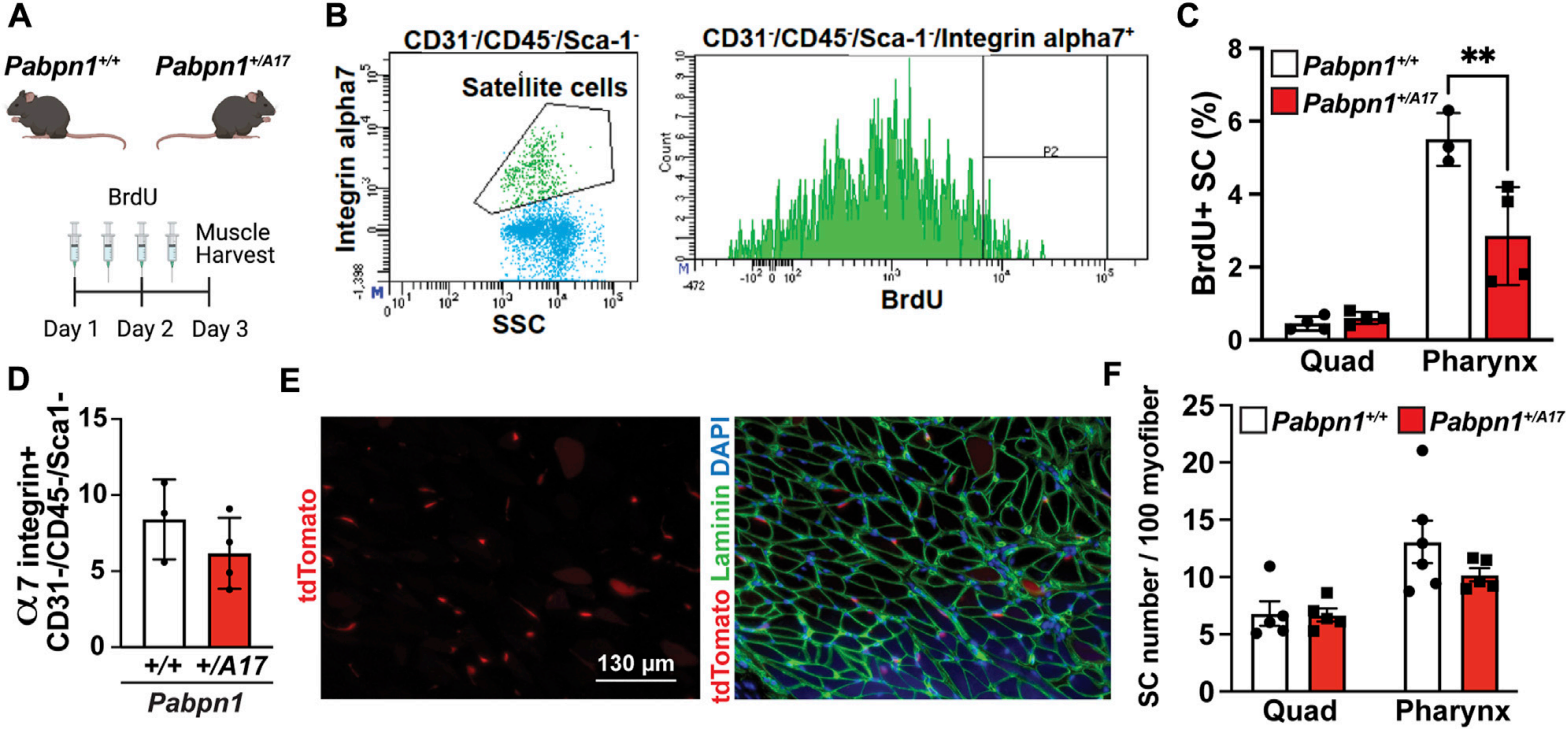
Proliferation of pharyngeal satellite cells is impaired in *Pabpn1* ^*+/A17*^ mice **(A)** Schematic of 2-day bromodeoxyuridine (BrdU) injection in *Pabpn1* ^*+/+*^ and *Pabpn1* ^*+/A17*^ mice. **(B)** Representative scatter plot (left) showing gating strategy to identify satellite cells as CD31^−^/CD45^−^/Sca1^−^/α7-integrin^+^ and histogram (right) showing gating strategy for BrdU^+^ satellite cells. **(C)** BrdU^+^ cells as percentage of total satellite cells (SC) revealing decreased BrdU^+^ SC in pharynx from *Pabpn1* ^*+/A17*^ mice but no change in quadriceps muscles (Quad). **(D)** Total number of pharyngeal CD31^−^/CD45^−^/Sca1^−^/α7-integrin^+^ satellite cells as a percentage of mononucleated cells showing no change in *Pabpn1* ^*+/A17*^ mice. **(E)** Representative cross-sectioned images of cricopharyngeus muscle showing tdTomato positive satellite cells in isolation (left) or merged with laminin-stained myofibers (right). Bar = 130 µm. **(F)** Number of tdTomato positive satellite cells (SC) per 100 myofibers quantified from sectioned quadriceps (Quad) and cricopharyngeus (Pharynx) muscles showing no change in *Pabpn1* ^*+/A17*^ mice. Shown is mean ± SEM for *n* = 3–5 mice. Statistical significance was determined using two-way ANOVA (** *p* < 0.01).

Decreased satellite cell fusion could be as a result of impaired fusion or overall decrease in satellite cell proliferation. Previously we reported the high level of pharyngeal satellite cells proliferation without injury. To determine if high level of basal satellite cell proliferation in pharyngeal muscles is affected in *Pabpn1* ^*+/A17*^ mice, we used bromodeoxyuridine (BrdU) to label nuclei of proliferating cells. *Pabpn1* ^*+/A17*^ and corresponding *Pabpn1* ^*+/+*^ mice were injected with BrdU for 2 days prior to muscle harvest and digestion (Figure 4A). Satellite cells isolated from pharyngeal and quadriceps muscles were identified by flow cytometry using known surface markers CD31^-^/CD45^-^/Sca1^-^/α7-integrin^+^ (Randolph et al., 2015) and proliferating satellite cells were identified as BrdU^+^ (Figure 4B). *In vivo* proliferation of pharyngeal satellite cells was reduced by half in *Pabpn1* ^*+/A17*^ mice when compared to *Pabpn1* ^*+/+*^ controls (Figure 4C). As expected, satellite cell proliferation was increased in pharyngeal muscle relative to quadriceps muscle (Figure 4C). The percentage of CD31^−^/CD45^−^/Sca1^−^/α7-integrin^+^ mononucleated cells was the same in pharyngeal muscles from *Pabpn1* ^*+/A17*^ and *Pabpn1* ^*+/+*^ mice (Figure 4D), suggesting that overall number of satellite cells does not change in *Pabpn1* ^*+/A17*^ mice. A previous study demonstrated that BrdU injection inhibited proliferation of RG2 glioma cells in rats (Levkoff et al., 2008). However, the dosage we used was approximately one-third of that used in rats. Nevertheless, to confirm that satellite cell number does not change, we counted tdTomato positive satellite cells in sectioned pharyngeal and quadriceps muscles from six-month-old *Pabpn1* ^*+/A17*^ x *Pax7-Cre* ^*ERT2*^ *-tdTomato* and *Pabpn1* ^*+/+*^ x *Pax7-Cre* ^*ERT2*^ *-tdTomato* mice 14 days after tamoxifen injection. Satellite cells labeled with tdTomato were detected inside of laminin-stained myofibers in cross-sectioned cricopharyngeal and rectus femoris muscles (Figure 4E). The number of satellite cells per 100 myofibers was significantly higher in cricopharyngeal versus quadriceps muscle, which is consistent with our previous studies (Randolph et al., 2015; Kim et al., 2022). However, there was no difference detected in the number of tdTomato positive satellite cells per 100 myofibers when comparing *Pabpn1* ^*+/A17*^ x *Pax7-Cre* ^*ERT2*^ *-tdTomato* and *Pabpn1* ^*+/+*^ x *Pax7-Cre* ^*ERT2*^ *-tdTomato* mice (Figure 4F). To determine if decreased proliferation of pharyngeal satellite cells in *Pabpn1* ^*+/A17*^ mice is associated with increased differentiation, we performed immunostaining for MyoD. However, no MyoD^+^ cells were detected in pharyngeal muscle sections from *Pabpn1* ^*+/+*^ or *Pabpn1* ^*+/A17*^ mice (data not shown), which is consistent with our previous studies showing no or very low MyoD in pharyngeal satellite cells by immunostaining (Kim et al., 2022) or qRT-PCR (Randolph et al., 2015). Taken together, these data indicate that the pharyngeal satellite cell pool is maintained in pharyngeal and limb muscles in *Pabpn1* ^*+/A17*^ mice while satellite cell proliferation is reduced in pharyngeal muscles only. These results agree with a previous study demonstrating impaired proliferation and myotube formation in myoblasts isolated from cricopharyngeal muscles of OPMD patients (Perie et al., 2006).

These data suggest a pharynx-specific defect in satellite cell activity in *Pabpn1* ^*+/A17*^ mice. Nevertheless, changes in basal limb satellite cell activity may be difficult to detect compared to the high rate of satellite cell proliferation and fusion in the basal state in pharyngeal and other craniofacial muscles (Pawlikowski et al., 2015; Randolph et al., 2015; Kim et al., 2022). To determine if satellite cell activity is impaired upon stimulation in limb muscle, we induced injury in tibialis anterior muscles using intramuscular BaCl_2_ injection (Supplementary Figure S2A). Seven days after injury, no regeneration defect was detected in *Pabpn1* ^*+/A17*^ mice (Supplementary Figures S2B,C) and regenerated myofiber cross-sectional area was actually slightly larger in *Pabpn1* ^*+/A17*^ mice relative to *Pabpn1* ^*+/+*^ controls (Supplementary Figure S2C). The slight increase could be a result of stronger satellite cell activity, which would be consistent with our previous study demonstrating increased central nucleated fibers in TA muscles from 6-month-old *Pabpn1* ^*+/A17*^ mice (Vest et al., 2017). Indeed, we previously reported that myofiber cross-sectional area in TA muscles from 18-month-old *Pabpn1* ^*+/A17*^ mice was increased relative to controls, suggesting that in limb muscles, satellite cells are more active and are not depleted over time. Thus, the satellite cell defect detected in *Pabpn1* ^*+/A17*^ mice is specific to pharyngeal muscles relative to limb muscles.

### Basal autophagy is impaired in pharyngeal myoblasts from *Pabpn1* ^*+/A17*^ mice

**FIGURE 5:**
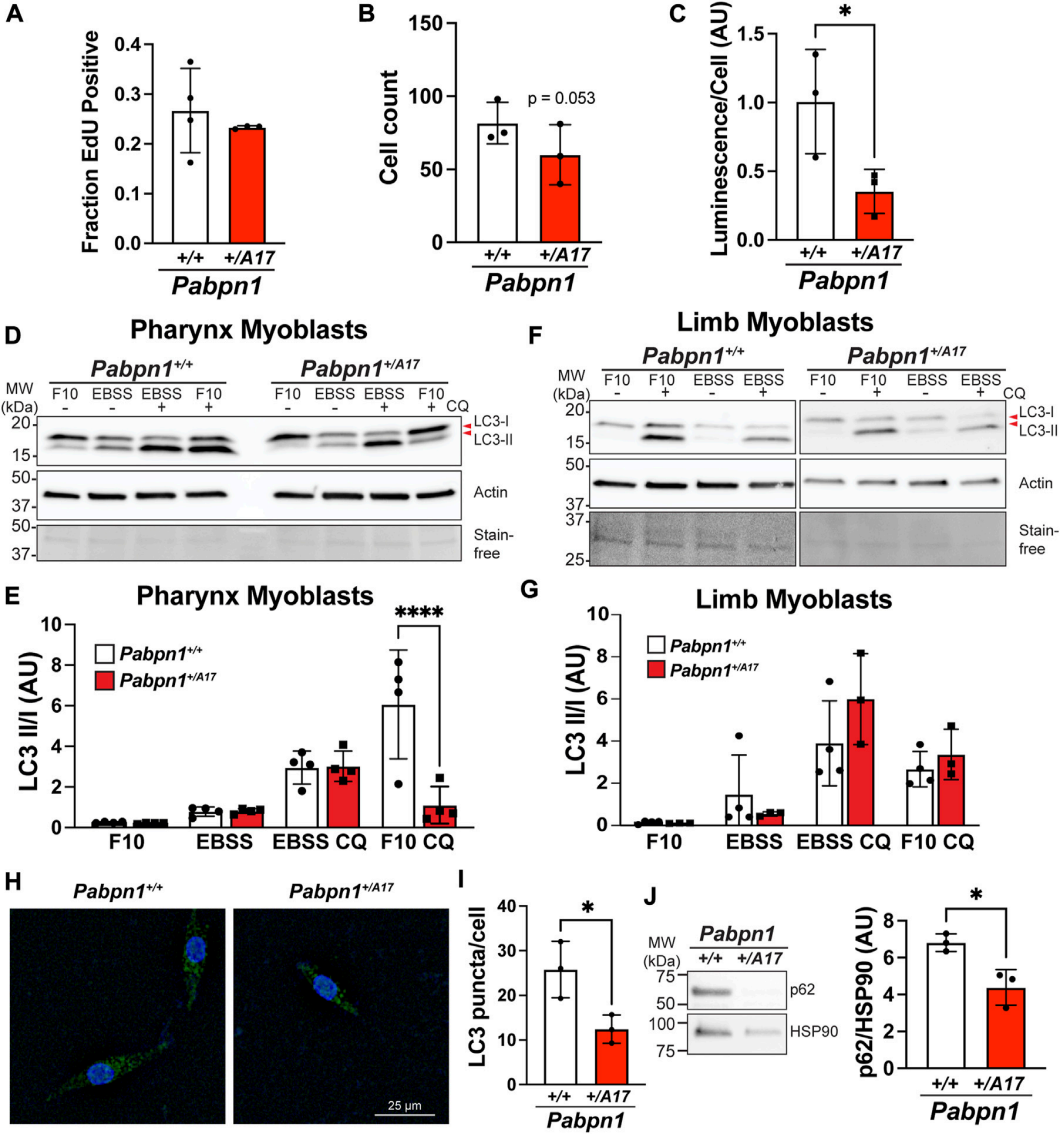
Impaired basal autophagy in pharyngeal but not limb myoblasts isolated from *Pabpn1* ^*+/A17*^ mice. **(A)** Fraction of EdU positive cells showing no significant difference in pharyngeal myoblasts from *Pabpn1* ^*+/A17*^ relative to *Pabpn1* ^*+/+*^ mice. **(B)** Total number of cells as quantified by counting DAPI-stained nuclei showing a trend toward fewer total cells in *Pabpn1* ^*+/A17*^ relative to *Pabpn1* ^*+/+*^ mice. **(C)** Cell viability reported as Luminescence per cell revealing a significant decrease in viable cells in pharyngeal myoblasts from *Pabpn1* ^*+/A17*^ compared to *Pabpn1* ^*+/+*^ mice. **(D)** Immunoblots to assay autophagy in pharyngeal myoblasts from *Pabpn1* ^*+/+*^ and *Pabpn1* ^*+/A17*^ mice grown in normal growth medium (F10) or under starvation conditions (EBSS) with or without the autophagosome-lysosome fusion inhibitor chloroquine (CQ). Blots were probed with an antibody to LC3-B to detect LC3-I and LC3-II, an antibody to β-actin (Actin) as a negative control, and imaged using stain free technology (Stain-free) as a loading control. **(E)** Quantification of blot shown in D revealing a significant decrease in *Pabpn1* ^*+/A17*^ myoblasts in F10 + CQ conditions. **(F)** Immunoblots quantifying autophagy in limb myoblasts. **(G)** Quantification of immunoblot shown in F revealing no difference in *Pabpn1* ^*+/+*^ and *Pabpn1* ^*+/A17*^ myoblasts. **(H)** Representative image of *Pabpn1* ^*+/+*^ and *Pabpn1* ^*+/A17*^ pharyngeal myoblasts immunostained with an antibody to LC3. Bar = 25 µm. **(I)** Quantification immunostain for LC3 revealing a significant decrease in LC3 puncta per cell in *Pabpn1* ^*+/A17*^ pharyngeal myoblasts. **(J)** Representative immunoblot and quantification p62 protein in pharyngeal myoblasts grown in F10 medium and treated with CQ revealing a significant decrease in p62 in *Pabpn1* ^*+/A17*^ cells. Immunoblot probed with an antibody to HSP90 was used as a loading control. Shown is mean ± SEM for *n* = 3–4 mice. Statistical significance was determined using t-test or one-way ANOVA (**** *p* < 0.0001, * *p* < 0.05).

To better understand the cell-intrinsic effects of expanded PABPN1 on satellite cells in *Pabpn1* ^*+/A17*^ mice, we isolated primary myoblasts from pharyngeal muscles and assayed cell proliferation and survival in culture. Using EdU to measure cell proliferation, we did not detect a significant difference between pharyngeal myoblasts from *Pabpn1* ^*+/A17*^ and *Pabpn1* ^*+/A17*^ mice (Figure 5A). However, we consistently noticed fewer cells growing in *Pabpn1* ^*+/A17*^ plates, as reflected by the trend (*p* = 0.053) toward decreased cell counts in *Pabpn1* ^*+/A17*^ pharyngeal myoblasts (Figure 5B). To determine if decreased cell counts were related to dying cells, we performed a luciferase-based viability assay and detected a significant decrease in viability as determined by decreased luminescence per cell (Figure 5C). Taken together, these data suggest the presence of expanded PABPN1 in *Pabpn1* ^*+/A17*^ mice causes cell-intrinsic defects in pharyngeal myoblasts.

Macroautophagy, known as autophagy, is the process by which cells degrade organelles and large protein complexes to recycle metabolites, remove damaged organelles, and promote cell proliferation and survival (Mathiassen et al., 2017). Autophagy is required for optimal activation of satellite cells and basal autophagy increases as satellite cells progress from quiescent to activated and from activated to the proliferating myoblasts stage (Tang and Rando, 2014; Fiacco et al., 2016; Garcia-Prat et al., 2016). Furthermore, loss of autophagy led to premature senescence and loss of satellite cells in the context of murine satellite cell maintenance (Garcia-Prat et al., 2016) and in human fibroblasts (Kang et al., 2011). Given that some studies have suggested that autophagy may be impaired in OPMD (Davies et al., 2006; Argov et al., 2016; Raz et al., 2017), we reasoned that autophagy may be impaired in *Pabpn1* ^*+/A17*^ mice. During autophagy, the microtubule associated protein light chain 3 (LC3) is conjugated to phosphatidylethanolamine (LC3-II) and recruited to the autophagosomal membrane (Jiang and Mizushima, 2015). Thus, we measured the ratio of LC3-II to LC3-I (unmodified) in myoblasts isolated from pharyngeal or limb muscles grown in normal growth medium (F10) or incubated in buffer (EBSS) to induce starvation (Figure 5D). Because LC3-II binds autophagosomes and is subsequently degraded (autophagic flux), it is difficult to determine the effects of genotype on autophagy by measuring LC3-II/I ratio alone (Mizushima and Yoshimori, 2007; Gottlieb et al., 2015). Therefore, we also analyzed myoblasts treated with chloroquine (F10 + CQ, EBSS + CQ), an inhibitor of autophagosome-lysosome fusion (Mauthe et al., 2018). We detected no change in LC3-b II/I ratio in pharyngeal myoblasts under normal growth medium or starvation conditions (Figure 5E). However, we noticed a strong and significant decrease in the LC3-b II/I ratio in *Pabpn1* ^*+/A17*^ pharyngeal myoblasts incubated in growth medium with the addition of chloroquine (Figure 5E). This result suggests that basal autophagosome formation is impaired in the presence of alanine-expanded (A17) PABPN1 but that defect is overcome when autophagy is induced by starvation. To determine if impaired autophagy is unique to pharyngeal myoblasts, we performed similar experiments in limb myoblasts and detected no defect (Figures 5F,G). Interestingly, *Pabpn1* ^*+/+*^ pharyngeal myoblasts had much higher basal autophagy compared to *Pabpn1* ^*+/+*^ limb myoblasts (Supplementary Figure S3A). Similar to limb myoblasts, no defect was detected in myotubes differentiated from pharyngeal myoblasts (Supplementary Figures S3B,C). To confirm the basal autophagy defect in pharyngeal myoblasts, we incubated pharyngeal myoblasts in normal growth medium plus chloroquine and immunostained for LC3 puncta (Figure 5H). We found that the number of puncta per cell was significantly lower in pharyngeal myoblasts from *Pabpn1* ^*+/A17*^ mice (Figure 5I). We also detected a significant decrease in the levels of p62/SQSTM1, another marker of autophagy, in pharyngeal myoblasts incubated in growth medium plus chloroquine (Figure 5J). The p62 marker is degraded upon autophagosome-lysosome fusion, so decreased p62 levels are associated with increased autophagy flux. Given that pharyngeal myoblasts were treated with chloroquine to inhibit phagosome-lysosome fusion, we would not expect this decrease to reflect increased autophagy. When we measured levels of p62 in pharyngeal myoblasts grown under basal conditions, we detected a significant increase in *Pabpn1* ^*+/A17*^ cells, which is consistent with decreased autophagy flux (Supplementary Figure 3D). Indeed, we discovered that under normal growth conditions, addition of chloroquine to *Pabpn1* ^*+/+*^ cells led to an increase in p62 levels while no change was observed in *Pabpn1* ^*+/A17*^ cells (Supplementary Figure 3E). Taken together, these data suggest a pharyngeal myoblast-specific autophagy defect.

**FIGURE 6:**
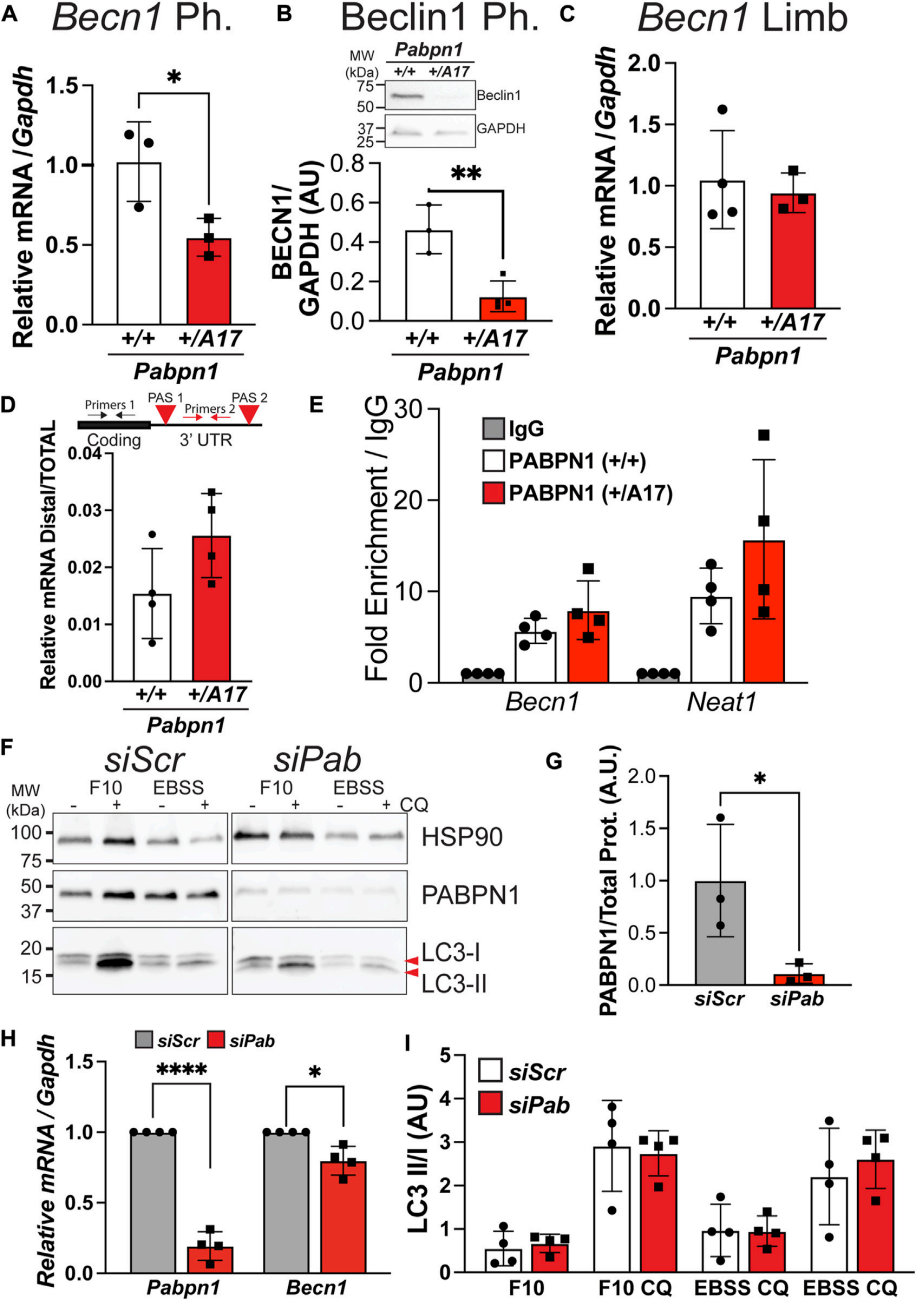
Impaired autophagy and low Becn1 RNA levels are not associated with loss of PABPN1 function in pharyngeal myoblasts from *Pabpn1* ^*+/A17*^ mice. **(A)** Steady-state levels of *Becn1* RNA determined by qRT-PCR showing a significant decrease in pharyngeal (Ph.) myoblasts from *Pabpn1* ^*+/A17*^ mice. **(B)** Levels of Beclin 1 protein as determined by immunoblot show a significant decrease in pharyngeal myoblasts from *Pabpn1* ^*+/A17*^ mice. GAPDH was used as a loading control. **(C)** Steady-state levels of *Becn1* RNA determined by qRT-PCR showing no change in limb myoblasts from *Pabpn1* ^*+/A17*^ mice. **(D)** Alternative polyadenylation as measured by qRT-PCR with primers designed to the distal 3′ untranslated region (UTR) between the two polyadenylation signals (PAS) as normalized to primers designed to amplify total steady-state *Becn1* RNA. No significant change was detected in pharyngeal myoblasts from *Pabpn1* ^*+/A17*^ mice. **(E)** RNA immunoprecipitation using an antibody to PABPN1 and lysates from pharyngeal myoblasts from *Pabpn1* ^*+/+*^ and *Pabpn1* ^*+/A17*^ mice. In both cases, *Becn1* binding to PABPN1 was detected by qRT-PCR and *Neat1* was used as a positive control. **(F)** Immunoblot of pharyngeal myoblasts treated with non-targeting siRNAs (*siScr*) or siRNA targeting *Pabpn1* (*siPab*). Control and knockdown cells were grown in normal growth medium (F10) or starvation conditions (EBSS) with or without chloroquine (CQ). Blots were probed with antibodies to LC3 to detect LC3-I and LC3-II, PABPN1 to detect knock down, and HSP90 as a negative control. **(G)** Quantification of PABPN1 levels from **(F)** showing ∼80% PABPN1 knockdown in siPab samples. **(H)** Steady-state levels of *Pabpn1* and *Becn1* in *siPab* normalized to *siScr* showing significant *Pabpn1* knockdown (left) and a small but significant decrease in *Becn1* RNA levels (right). **(I)** Quantification of LC3-II/I ratio from immunoblot in **(F)** showing no change in autophagy in *Pabpn1* knockdown cells. Shown is mean ± SEM for *n* = 3–4 mice or *Pabpn1* knockdown/*siScr* cells. Statistical significance was determined by t-test, one-way ANOVA, or two-way ANOVA (* *p* < 0.05, ** *p* < 0.01, **** *p* < 0.0001).

A previous study reported altered steady-state levels of RNAs encoding autophagy proteins in OPMD patient tissues and in muscle tissue from the A17.1 overexpression mouse model of OPMD (Raz et al., 2017). We measured steady-state levels of several transcripts encoding autophagy proteins including *Atg5*, *Atg10*, *Wipi*, and *Maplc3a* but did not detect any difference between *Pabpn1* ^*+/A17*^ pharyngeal myoblasts and *Pabpn1* ^*+/+*^ pharyngeal myoblasts (Supplementary Figure S4A). However, pharyngeal myoblasts from *Pabpn1* ^*+/A17*^ mice did show a significant decrease in the steady-state levels of *Becn1* RNA (Figure 6A) and protein (Figure 6B), which is a central component of early autophagosome formation (Cao and Klionsky, 2007). Interestingly, no change in *Becn1* levels was detected in limb myoblasts (Figure 6C).

One major function of PABPN1 is regulating alternative polyadenylation by masking weaker polyadenylation signals (PAS) in the 3′ untranslated region (UTR) (De Klerk et al., 2012; Jenal et al., 2012). A previous study showed altered PAS utilization in RNAs encoding autophagy proteins in A17.1 transgenic mice that correlated with altered steady-state levels in some of these RNAs (Raz et al., 2017). Like most mammalian transcripts, the *Becn1* RNA contains two PAS, so we sought to determine whether altered PAS utilization contributes to the low *Becn1* levels detected in pharyngeal myoblasts from *Pabpn1* ^*+/A17*^ mice. We designed primers to the distal 3’ UTR of *Becn1*, which is present only in transcripts that use the distal most PAS and used qPCR to compare distal primer product to the product from primers that target the total transcript pool. The ratio of distal transcripts to total transcripts was highly variable and there was no significant difference in PAS utilization (Figure 6D), though there was a slight trend toward more distal PAS-using transcripts in *Pabpn1* ^*+/A17*^ pharyngeal myoblasts. This result suggests that some other mechanism drives the decreased Becn1 levels detected in *Pabpn1* ^*+/A17*^ pharyngeal myoblasts. To confirm that *Becn1* RNA is a target of PABPN1, we performed immunoprecipitation using an antibody to PABPN1 and RNA isolation and qPCR to measure levels of *Becn1* RNA captured with PABPN1. We found that the PABPN1 antibody precipitated *Becn1* RNA and the positive control *Neat1* RNA in both *Pabpn1* ^*+/+*^ and *Pabpn1* ^*+/A17*^ pharyngeal myoblasts (Figure 6E). This result suggests that alanine expansion does not impede RNA binding and is consistent with existing literature suggesting that expanded PABPN1 stimulates poly(A) polymerase activity to a similar degree as wild type PABPN1 (Banerjee et al., 2013).

Decreased availability of PABPN1 may contribute to the lower levels of *Becn1* RNA. We measured steady-state levels of *Pabpn1* RNA (Supplementary Figure S5A) and PABPN1 protein (Supplementary Figures S5B,C) in *Pabpn1* ^*+/A17*^ pharyngeal myoblasts but detected no difference relative to PABPN1 in *Pabpn1* ^*+/+*^ pharyngeal myoblasts. To determine if loss of PABPN1 function causes impaired autophagy in pharyngeal myoblasts, we used siRNA to knock down *Pabpn1* in wild type pharyngeal myoblasts (Figures 6F,G). Although we detected a small but significant decrease in *Becn1* RNA (Figure 6H) when Ct values from *siPab* samples were normalized to Ct values from *siScr* samples, there was no significant difference when compared to non-transfected cells (Supplementary Figure S6A) and no defect in autophagosome formation was detected in *Pabpn1* knockdown cells (Figure 6I). Previous studies have suggested that a combination of exogenous recombinant PABPN1 and endogenous *Pabpn1* knockdown is sufficient to rescue muscle phenotypes in A17.1 mice (Malerba et al., 2017; Abu-Baker et al., 2019; Malerba et al., 2019a). We transfected *Pabpn1* ^*+/+*^ and *Pabpn1* ^*+/A17*^ pharyngeal myoblasts with plasmids encoding FLAG-tagged human PABPN1 (FL-hsPABPN1) (Banerjee et al., 2019) or FL-hsPABPN1 along with siRNA targeting Exon 7 of murine *Pabpn1*. We incubated transfected cells with chloroquine and then analyzed LC3 II/I ratios by immunoblot. We found that expression of FL-hsPABPN1 alone was not sufficient to restore the LC3 II/I ratio in *Pabpn1* ^*+/A17*^ pharyngeal myoblasts to wild type levels (Supplementary Figures S6B,C). Surprisingly, the murine-targeted *Pabpn1* siRNA inhibited expression of FL-hsPABPN1, but the protein was still detectable (Supplementary Figure S6B). Although there appeared to be no difference in LC3 II/I ratio between *Pabpn1* ^*+/+*^ and *Pabpn1* ^*+/A17*^ pharyngeal myoblasts, the variability of the data resulted in none of the comparisons being significant (Supplementary Figure S6C). However, a trend toward lower LC3 II/I in *Pabpn1* ^*+/A17*^ pharyngeal myoblasts was not detected upon Pabpn1 knockdown and expression of FL-hSPABPN1. (Supplementary Figure S6C) Taken together, these data suggest that PABPN1 levels are normal in *Pabpn1* ^*+/A17*^ pharyngeal myoblasts and that loss of PABPN1 function does not result in impaired autophagy.

## Discussion

In this study, we sought to understand the pharyngeal muscle pathology in a *Pabpn1* ^*+/A17*^ knock-in mouse model of OPMD. Unlike studies in previous mouse models, we detected both functional and histologic pathology in pharyngeal muscles of *Pabpn1* ^*+/A17*^ mice. Mild dysphagia as detected by lick assay along with a small decrease in cricopharyngeal myofiber cross-sectional area was detected in six-month-old mice, which suggests that this time point can be compared to the early stages of disease in OPMD. Lick assays performed at 12 months of age did not reveal any decrease in *Pabpn1* ^*+/A17*^ mice suggesting that this model is not appropriate for studying the progression of OPMD. Interestingly, we detected impaired satellite cell proliferation and fusion in *Pabpn1* ^*+/A17*^ mice *in vivo* and impaired cell survival and basal autophagy in satellite cell-derived cultured myoblasts from *Pabpn1* ^*+/A17*^ mice *in vitro*. The proliferation and autophagy defects were detected only in cells in or derived from pharyngeal muscle, suggesting that muscle-specific but cell intrinsic differences in satellite cell function may contribute to OPMD. Loss of PABPN1 function by siRNA-mediated knockdown in pharyngeal myoblasts did not impair autophagy, suggesting that aberrant function of Ala17-PABPN1 contributes to the defects detected in *Pabpn1* ^*+/A17*^ mice. These results elaborate on previous studies in tissues isolated from individuals with OPMD that suggest aberrant pharyngeal satellite cell function and impaired autophagy and further highlight the utility of the *Pabpn1* ^*+/A17*^ mouse model of OPMD.

This study is the first to demonstrate a functional pharyngeal muscle defect in a mouse model of OPMD. Our data suggest a small but significant functional pharyngeal muscle deficit in *Pabpn1* ^*+/A17*^ mice as demonstrated by lick assay, a measurement for swallow function. A previous study found that muscle-specific over expression of wild type PABPN1 but not Ala17 PABPN1 improved age-related dysphagia as detected by lick assay (Randolph et al., 2014). However, no direct defect was detected from muscle specific over expression of Ala17 PABPN1 alone. Interestingly, even though no pharyngeal muscle defects were detected in A17.1 mice, severe limb muscle functional deficits were observed (Davies et al., 2005), which we did not detect in *Pabpn1* ^*+/A17*^ mice. The lack of pharyngeal phenotype in mouse model that express expanded PABPN1 only in mature myofibers suggests that other cell types may be relevant in the pharyngeal pathology of OPMD.

Indeed, we detected a significant decrease in pharyngeal muscle satellite cell proliferation and fusion in *Pabpn1* ^*+/A17*^ mice. This result suggests that impaired satellite cell proliferation may contribute to pharyngeal muscle dysfunction. These results agree with a previous study in OPMD patients that revealed slightly higher numbers of satellite cells in cricopharyngeal muscles, but rapid proliferative arrest and loss of myotube formation when those cells were grown in culture (Perie et al., 2006). Additionally, we recently reported that high numbers of PDGFRα^+^ fibroadipogenic progenitor cells (FAPs) in pharyngeal muscles contribute to pharyngeal SC basal proliferation (Kim et al., 2022) and that ablation of pharyngeal FAPs impairs satellite cell proliferation to a similar extent as the presence of alanine expanded PABPN1 in *Pabpn1* ^*+/A17*^ mice. In FAPs ablated muscle, however, pharyngeal muscle function was much more severely impacted than it was in *Pabpn1* ^*+/A17*^ mice. This comparison suggests that satellite cells and FAPs make distinct contributions to pharyngeal muscle function. Interestingly, H&E-stained sections from injured TA muscles suggest the presence of fibrosis in *Pabpn1* ^*+/A17*^ mice, which may be a result of aberrant FAPs activity. Thus, future studies are needed to determine the functional effects of expanded PABPN1 in FAPs in limb and pharyngeal muscles.

Satellite cell activation and proliferation depend on activation of autophagy, likely to provide metabolites for mounting anabolic and catabolic demands during activation, proliferation, and fusion (Tang and Rando, 2014; Fiacco et al., 2016; Garcia-Prat et al., 2016). Indeed autophagy contributes to proliferation and cell survival in multiple cell types (Kang et al., 2011; Das et al., 2012). We studied basal and starvation-induced autophagy in myoblasts derived from pharyngeal and limb muscles. Interestingly, we detected a significant defect in basal autophagy in pharyngeal myoblasts that was not detected in limb myoblasts or myotubes differentiated from pharyngeal or limb myoblasts. This result suggests that the presence of alanine-expanded PABPN1 negatively influences autophagy and highlights the existence of cell-intrinsic defects in pharyngeal satellite cells in *Pabpn1* ^*+/A17*^ mice. We found that expression of exogenous tagged wild type (A10) PABPN1 was not sufficient to rescue the basal autophagy defect in *Pabpn1* ^*+/A17*^ pharyngeal myoblasts. Cells co-transfected with wild type PABPN1 and *Pabpn1*-targeting siRNA appeared to be partially rescued, suggesting that the previously reported knockdown and replace strategy would be viable in targeting OPMD-associated satellite cell pathology. However, additional future studies are needed to resolve the contributions of satellite cell-intrinsic effects versus niche factor effects to the pharyngeal pathology in OPMD.

A small but significant decrease in *Becn1* RNA, which encodes the master regulator of autophagy, was detected in pharyngeal myoblasts from *Pabpn1* ^*+/A17*^ mice, suggesting that loss of Beclin1 function may drive the basal autophagy defect that is overcome by inducing starvation conditions. PABPN1 is known to regulate alternative polyadenylation and cleavage, but only detected a small trend toward altered alternative polyadenylation in *Becn1* RNA. Therefore, alternate mechanisms likely contribute to the decrease in *Becn1* in pharyngeal myoblasts. Interestingly, *Pabpn1* knockdown in pharyngeal myoblasts did not impair basal autophagy or decrease *Becn1* RNA levels. This result suggests that loss of PABPN1 function does not drive an autophagy defect and is consistent with the fact that no difference in PABPN1 binding to *Becn1* RNA was detected in *Pabpn1* ^*+/A17*^ pharyngeal myoblasts compared to controls. Taken together, these results suggest that alanine expansion imparts an aberrant gain of function to PABPN1 in the context of pharyngeal myoblasts. Alanine expansion in PABPN1 is associated with nuclear aggregate formation but aggregates are not detected in myoblasts. Alternatively, expanded PABPN1 may form pre-aggregate structures or aberrant complexes with other RNA binding proteins that negatively impact *Becn1* RNA. Indeed, our previous proteomic study revealed that alanine expanded PABPN1 forms higher molecular weight complexes binds to a different and larger complement of protein binding partners (Banerjee et al., 2019). These studies were performed in murine limb muscle and were also confounded by the fact that both wild type and expanded PABPN1 were overexpressed, which may impact the function of PABPN1. In the future, detailed comparison of PABPN1 and expanded PABPN1 protein-protein interactions may identify the aberrant complexes that drive limb versus pharyngeal muscle pathology. These studies may finally identify the mechanistic details behind OPMD pathology and lead to development of new therapeutic options for affected individuals.

## Data Availability

The original contributions presented in the study are included in the article/Supplementary Material; further inquiries can be directed to the corresponding authors.
