# Supplementary material for: Pharyngeal pathology in a mouse model of oculopharyngeal muscular dystrophy is associated with impaired basal autophagy in myoblasts
Source: Front Cell Dev Biol. 2022 Oct 14;10:986930. doi: 10.3389/fcell.2022.986930 (PMC9614327; doi:10.3389/fcell.2022.986930)
Supplement: Supplementary file 1 [file DataSheet1.PDF]

FIGURE S1

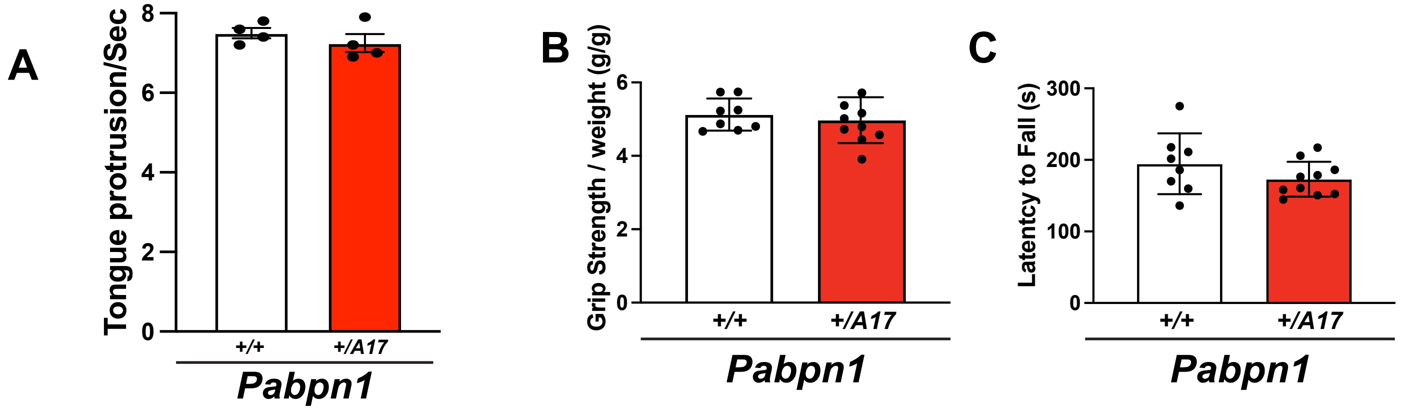

Figure S1: No age-related pharyngeal pathology and no functional limb muscle pathology detected in *Pabpn1*$^{+/A17}$ mice *(A)* Number of tongue protrusions per second does not change in 12-month-old *Pabpn1*$^{+/A17}$ mice as counted by video analysis of lick assay. ***(B)*** Forelimb grip strength (two paws) normalized to total body weight (g/g) with no change detected in 6-month-old *Pabpn1*$^{+/A17}$ mice. ***(C)*** Rotarod latency to fall (s) with no change detected in 6-month-old *Pabpn1*$^{+/A17}$ mice. Shown is mean ± SEM for n = 8-9 mice.

FIGURE S2

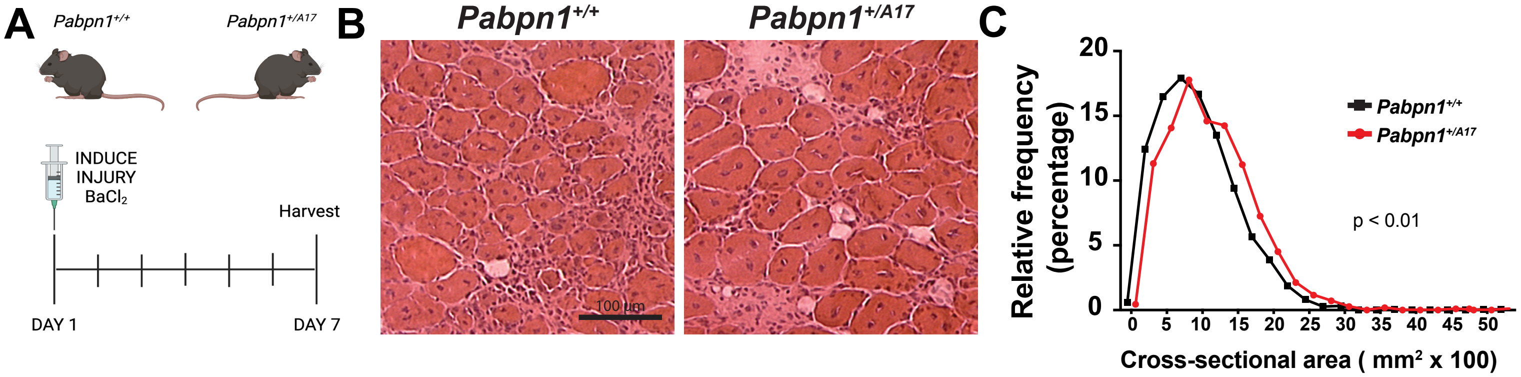

Figure S2: *Pabpn1*$^{+/A17}$ mice do not exhibit a limb muscle regeneration defect ***(A)*** Schematic of seven-day barium chloride ($BaCl_2$) induced injury experiment performed in tibialis anterior muscles of *Pabpn1*$^{+/+}$ and *Pabpn1*$^{+/A17}$ mice. ***(B)*** Representative H&E-stained cross sections of tibialis anterior muscles from *Pabpn1*$^{+/+}$ and *Pabpn1*$^{+/A17}$ mice seven days after injury. Bar = 100 μm. ***(C)*** Frequency distribution of regenerated TA myofiber cross-sectional area (mm2) revealing a significant increase in regenerated myofiber size in *Pabpn1*$^{+/A17}$ mice. Regenerated myofibers were identified as central nucleated fibers. Shown is frequency distribution of binned cross-sectional area data from n = 3 mice per genotype. Statistical significance was determined using Kolmogorov-Smirnov test.

FIGURE S3

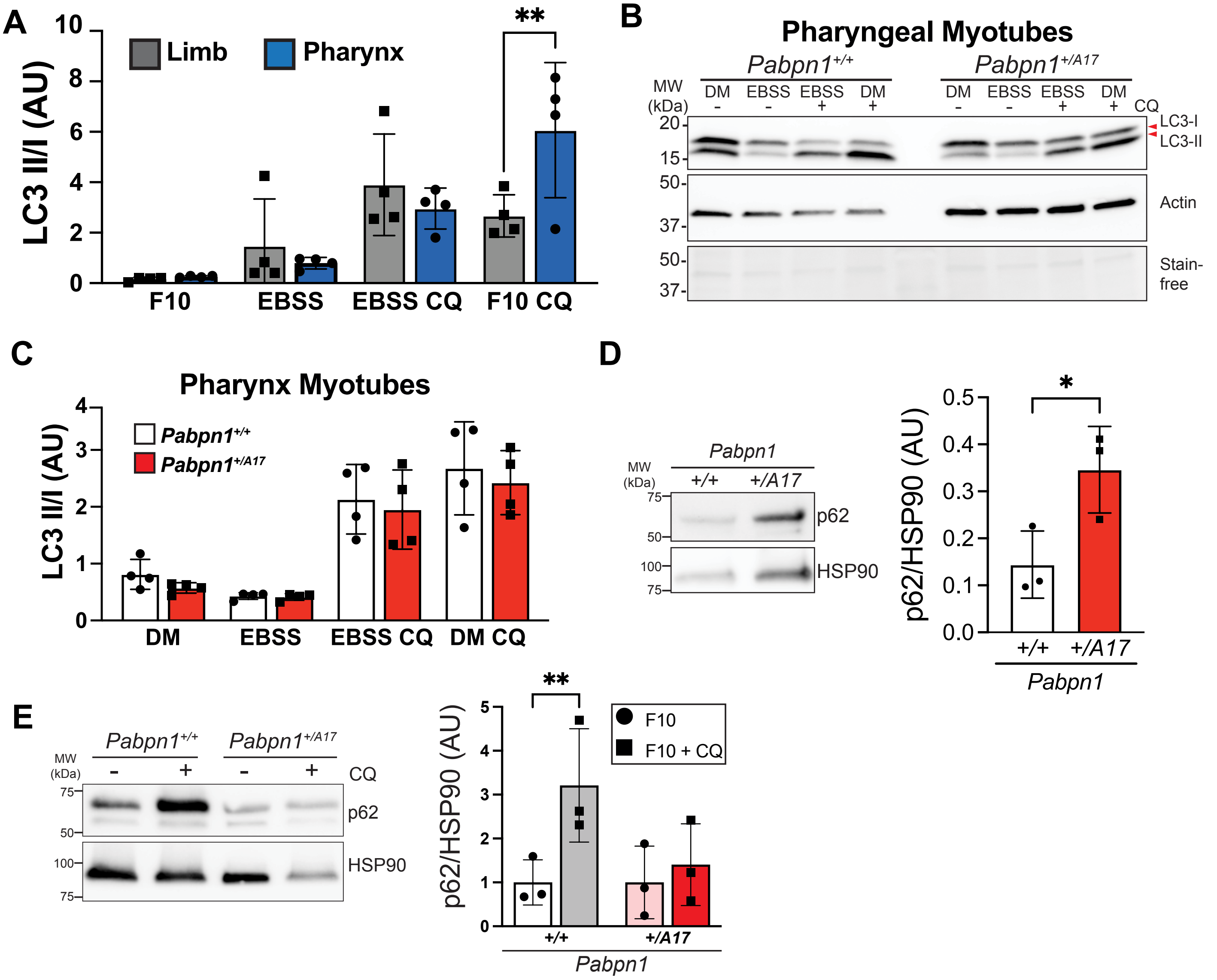

Figure S3: Increased basal autophagy in pharyngeal myoblasts relative to limb. (A) Quantification of immunoblots shown in Figure 5 D and F comparing pharyngeal versus limb myoblasts from *Pabpn1*$^{+/+}$ mice. **(B)** Immunoblot of pharyngeal myotubes subjected to starvation (EBSS) or kept in normal differentiation medium (DM) with or without chloroquine (CQ). **(C)** Quantification of immunoblot shown in B showing no change in pharyngeal myotubes from *Pabpn1*$^{+/A17}$ mice. **(D)** Immunoblot and quantification showing increased p62 levels in pharyngeal myoblasts from *Pabpn1*$^{+/A17}$ mice grown under normal growth conditions (F10). **(E)** Immuboblot and quantification comparing p62 levels from *Pabpn1*$^{+/+}$ and *Pabpn1*$^{+/A17}$ pharyngeal myoblasts grown in normal growth medium (F10) or treated with chloroquine (F10 + CQ) showing an increase in p62 levels in *Pabpn1*$^{+/+}$ but not *Pabpn1*$^{+/A17}$ cells after CQ treatment. Shown is mean ± SEM for n = 4 mice. Statistical significance was determined using one-way ANOVA (** $p < 0.01$. * $p < 0.05$).

FIGURE S4

A

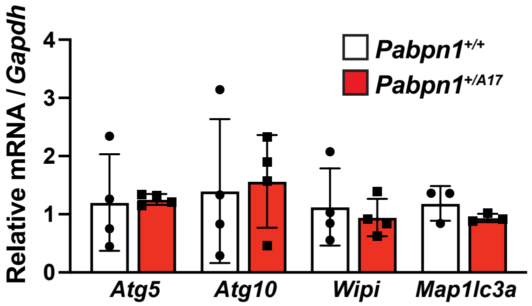

Figure S4: No change is detected in levels of markers of autophagy previously reported to be affected in OPMD muscles. Steady-state RNA levels of the markers for autophagy *Atg5, Atg10, Wipi*, and *Map1lc3a* as measured by qRT-PCR normalized to *Gapdh* show no change in pharygeal myoblasts from *Pabpn1*$^{+/A17}$ mice. Shown is mean ± SEM for n = 3-4 mice.

FIGURE S5

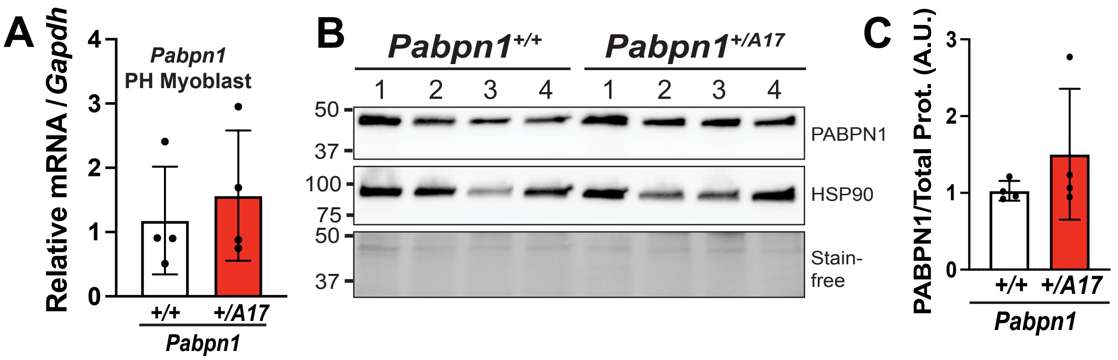

Figure S5: No change is detected in PABPN1 RNA or protein levels in pharyngeal myoblasts from *Pabpn1*$^{+/A17}$ mice. **(A)** No change in Pabpn1 RNA steady-state levels in pharyngeal myoblasts from *Pabpn1*$^{+/A17}$ mice as measured by qRT-PCR normalized to *Gapdh*. **(B)** Immunoblot of pharyngeal myoblasts from *Pabpn1*$^{+/+}$ and *Pabpn1*$^{+/A17}$ mice. Blots were probed with antibodies to PABPN1, HSP90 as a negative control and imaged using stain-free technology (Stain-free) as a loading control. **(C)** Quantification of PABPN1 levels (A.U) normalized to total protein from blot shown in *(C)* showing no change in PABPN1 protein levels in *Pabpn1*$^{+/A17}$ mice. Shown is mean ± SEM for n = 4 mice.

FIGURE S6

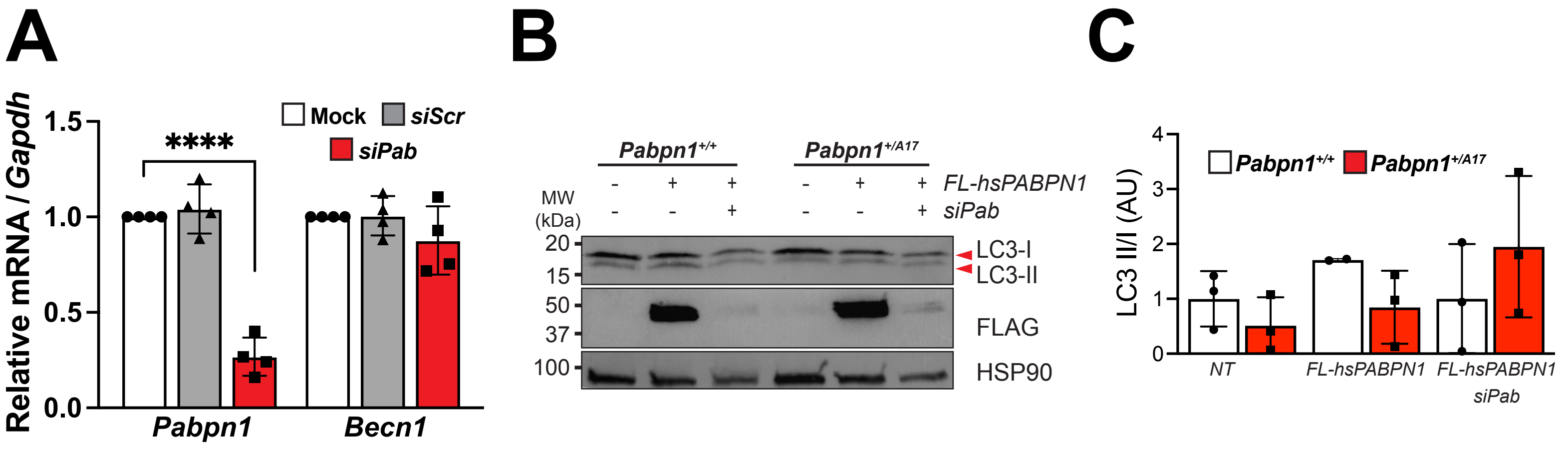

Figure S6: No change in *Becn1* RNA levels in *Pabpn1* knockdown cells when compared to mock transfected cells. **(A)** Steady-state levels of *Pabpn1* (left) and *Becn1* in pharyngeal myoblasts treated with transfection reagent alone (Mock), non-targeting siRNA (*siScr*), or siRNA targeting Pabpn1 (*siPab*). A significant decrease in *Pabpn1* but no change in *Becn1* is detected when normalized back to mock-transfected cells. **(B)** Immunoblot probing for LC3 II/I ratio in *Pabpn1*$^{+/+}$ and *Pabpn1*$^{+/A17}$ pharyngeal myoblasts transfected with either mock (NT), *FL-hsPABPN1*, or *FL-hsPABPN1* + murine *Pabpn1* targeting siRNA grown in normal growth medium and treated with chloroquine. **(C)** Quantification of immunoblot shown in B. Shown is mean ± SEM for n = 4. **** $p < 0.0001$.

Table S1: PCR Primers

Technique	Gene target/Experiement	Primer	Sequence (5'-3')
Standard PCR	Pabpn1/Genotyping	Shared F for WT and Ala17	GACTGACTAATGAATCCTCGGCG
		R primer for WT	GCCGCCATCGCCGCTCAGAC
		R primer for Ala17	TTCGTATAATGTATGCTATACGAAGTTATTTCGAAG
qRT-PCR	*Gapdh* normalizer	F	AAGGTCGGAGTCAACGGATTTGG
		R	GATGACAAGCTTCCCGTTCTC
	Becn1 levels and RIP	F	AGAGGCTAACTCAGGAGAGGAG
		R	TGTAGACATCATCCTGGCTGGG
	Becn1 Distal UTR for APA	F	TGCACAAACACTCGTGCG
		R	GCCATCAACACAGGAATCAGGA
	Neat1 levels and RIP	F	GATCGGGACCCCAGTGACCTC
		R	CAACAGCTTTCCCCAACACCCAC
	Pabpn1	F	AACAGACCAGGCATCAGCAC
		R	ATCGGGAGCTGTTGTAGTTGG
	Atg5	F	TGAAGGCACACCCCTGAAAT
		R	TGTTCCAAGGAAGAGCTGAACT
	Atg10	F	CTCAGCCAACCTGCAACTTT
		R	GACTTGCTTCAGAGTGCCCA
	Wipi	F	AGGCCGGTTACAAGCTGTTT
		R	AGGCGCTCCACGATATACAC
	Map1lc3a	F	TCCCCAGTGGATTAGGCAGA
		R	ACCCAAAAGAGCAACCCGAA

Table S2: Antibodies

Experiment	Antibody/target	Manufacturer	Clone/Catalog number	Dilution/Concentration
Flow cytometry	CD45-PE	BD Biosciences	Clone 30-F11	1:400
	CD31-PE	eBiosciences	Clone 390	1:400
	Sca1-PE-Cy7	BD Biosciences	Clone D7	1:4000
	a7-integrin-APC	AbLab	Clone R3F2	1:500
Immunostaining	Laminin	Sigma	Cat #L9393	1:300
	FITC-anti-rabbit	Jackson ImmunoResearch	Cat # 715-095-152	1:500
	LC3	Cell Signaling Technologies	Cat #2775	1:200
Immunoblotting	LC3	Cell Signaling Technologies	Cat #2775	1:1000
	PABPN1	abcam	EP3000Y	1:1000
	Beta-actin	Cell Signaling Technologies	8H10D10	1:8000
	HSP90	Cell Signaling Technologies	C45G5	1:4000
	p62	Cell Signaling Technologies	Cat #5114	1:1000
	GAPDH	Cell Signaling Technologies	Cat #5114	1:10,000
	Beclin1	Cell Signaling Technologies	Cat #3738	1:1000
	HRP-anti-mouse	Jackson ImmunoResearch	Cat # 111-035-003	1:5000
	HRP-anti-rabbit	Jackson ImmunoResearch	Cat # 115-035-003	1:5000
RIP	PABPN1	Cell Signaling Technologies	Cat #2775	1 μg
	Rabbit IgG	R & D Systems	Cat #AB-105-C	1 μg
